# Lightweight and High‐Strength Carbon Bulk Preparation Via Low‐Temperature Pressureless Sintering of Sugar‐Derived Carbon

**DOI:** 10.1002/advs.202518437

**Published:** 2025-11-21

**Authors:** Chenlin Hou, Daming Chen, Mingyi Tan, Wenzheng Zhang, Boqian Sun, Guiqing Chen, Wenbo Han, Ruiqun Pan, Shanyi Du, Jiecai Han, Xinghong Zhang

**Affiliations:** ^1^ National Key Laboratory of Science and Technology on Advanced Composites in Special Environments Harbin Institute of Technology Harbin 150001 P. R. China; ^2^ Suzhou Laboratory Suzhou 215000 P. R. China; ^3^ Jiangsu Mogui New Materials Co., Ltd. Changzhou 213127 P. R. China; ^4^ School of Material Science and Chemical Engineering Harbin University of Science and Technology Harbin 150001 P. R. China

**Keywords:** carbon bulks, glucose, hydrogels, mechanical properties, pressureless sintering

## Abstract

Bulk carbon materials are widely used in the fields of high‐temperature and corrosion resistance due to their excellent stability. The inertness of carbon structure obstructs the sintering process and encourages the excessive need for mesophase pitch, high pressure, and high temperature in the conventional carbon material industry. The development of a high‐strength, lightweight, and graphitizable sugar‐derived carbon bulk is discussed herein. The cracking issue caused by devolatilization of the organic ingredients is well dealt with through a customized sugar hydrogel pressureless sintering strategy. The sintering process is completed without applying pressure at 1200 °C, owing to the active nature induced by glucose. Macro‐sized bulk carbon artifacts with superior strengths (581.0 MPa compressive and 153.4 MPa flexural) and low bulk densities (≈1.28 cm^3^) are demonstrated. By carrying out the fabrication of carbon bulks using renewable glucose, this study provides valuable insights for the structural design and sustainable fabrication of future high‐performance carbon bulks.

## Graphical abstract

The synergistic effect between crosslinked polyacrylamide and glucose brings the precursor sintering activity with better thermostability, allowing its formation of a coherent carbon structure at lower temperatures (≤ 1200 °C) under ambient pressure. This combination of two types of carbon source generates a uniform carbon structure composed of few‐layer graphene and amorphous carbon, resulting in remarkable mechanical properties.

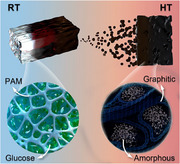

## 1 Introduction

Owing to their excellent mechanical strength, remarkable thermal and chemical stability, low density, and tunable electrical conductivity, carbon materials and their composites have found wide applications in various fields of material science.^[^ ^1^, ^2^, ^3^, ^4^, ^5^ ^]^ In the subtractive manufacturing industry, particularly, bulk carbon materials are constantly utilized as tool electrodes of electrical discharge machining (EDM).^[^ ^6^ ^]^ The EDM is a non‐contact, high‐precision technique that removes the workpiece efficiently regardless of its hardness.^[^ ^7^ ^]^ Compared to copper electrodes, carbon electrodes have advantages in weight, thermal stability, electrical resistance, and machinability, which brings higher efficiency, higher precision, and longer electrode service life.^[^ ^8^, ^9^, ^10^, ^11^ ^]^ However, the superior thermal stability of carbon materials comes with a price of poor diffusivity, which leads to poor sintering activity. Besides high temperature and high pressure, the conventional route for artificial graphite production excessively needs preprocessed pitch as a binder to integrate the filler (coke, mesocarbon microbeads (MCMBs), etc.) particles and create a coherent structure.^[^ ^4^, ^12^, ^13^ ^]^ However, the thick nature of pitch leads to poor interfacial bonding with the filler particles, and the volatiles generated from heated pitch leaves irregular pores inside of the carbon artifact, which badly jeopardize the mechanical strength of the products.^[^ ^14^, ^15^ ^]^ The overuse of high‐temperature heat treatment causes overgrowth of graphite crystals that exposes their weak interlayer bonding. As a result, the performances of the current products remain unsatisfactory, whose flexural strength hardly exceeds 100 MPa (Table S1, Supporting Information).

It has been reported that carbon bulks with better performances were obtained through spark plasma sintering (SPS) and chemical vapor deposition (CVD).^[^ ^16^, ^17^, ^18^, ^19^ ^]^ Nevertheless, the SPS technology has trouble in large‐scale fabrication, while the CVD process suffers from high cost and low efficiency, and both of them are still using non‐renewable carbon sources. Even with the advances in sintering technology, the sintering inertia of carbon material remains a challenge. With the growing awareness of environmental conservation, researchers have been working on producing carbon materials using renewable carbon sources derived from biomasses, and progress has been made in the fields of low‐dimensional and foam‐like carbon materials.^[^ ^20^, ^21^, ^22^, ^23^, ^24^, ^25^, ^26^, ^27^ ^]^ However, obstructed by the devolatilization behavior of organic substances under high temperature, little has been achieved in fabricating densified carbon bulk with biomass.^[^ ^28^, ^29^ ^]^ Among all the candidates from biomass, sugar (glucose, fructose, sucrose, etc.) seems to be a convenient choice with excellent fusibility that allows low‐temperature sintering. In our previous study, a densified carbon structure was obtained using sugar as the main carbon source through a sugar hydrogel pyrolysis (SHP) method, which presented remarkable performances in microscale but was flawed in macroscale.^[^ ^30^ ^]^ Based on the principle of the SHP method, materials including carbon foams, carbon fibers, highly oriented carbon films, and C/C composites with excellent properties were successfully obtained.^[^ ^22^, ^23^, ^24^, ^31^ ^]^ But when it comes to the fabrication of bulk carbon material, the conflict between the densification process and the devolatilization process creates tremendous internal stress that cracks the bulk during heat treatment. Despite the cracks generated in the pre‐carbonization process of the SHP method, it is noticed that the precursor body created from sugar hydrogel exhibits perfect shape stability with certain self‐sintering ability, suggesting a promising potential to achieve sintering while stabilizing the decomposition of sugar to avoid structural disintegration.

**Figure 1:**
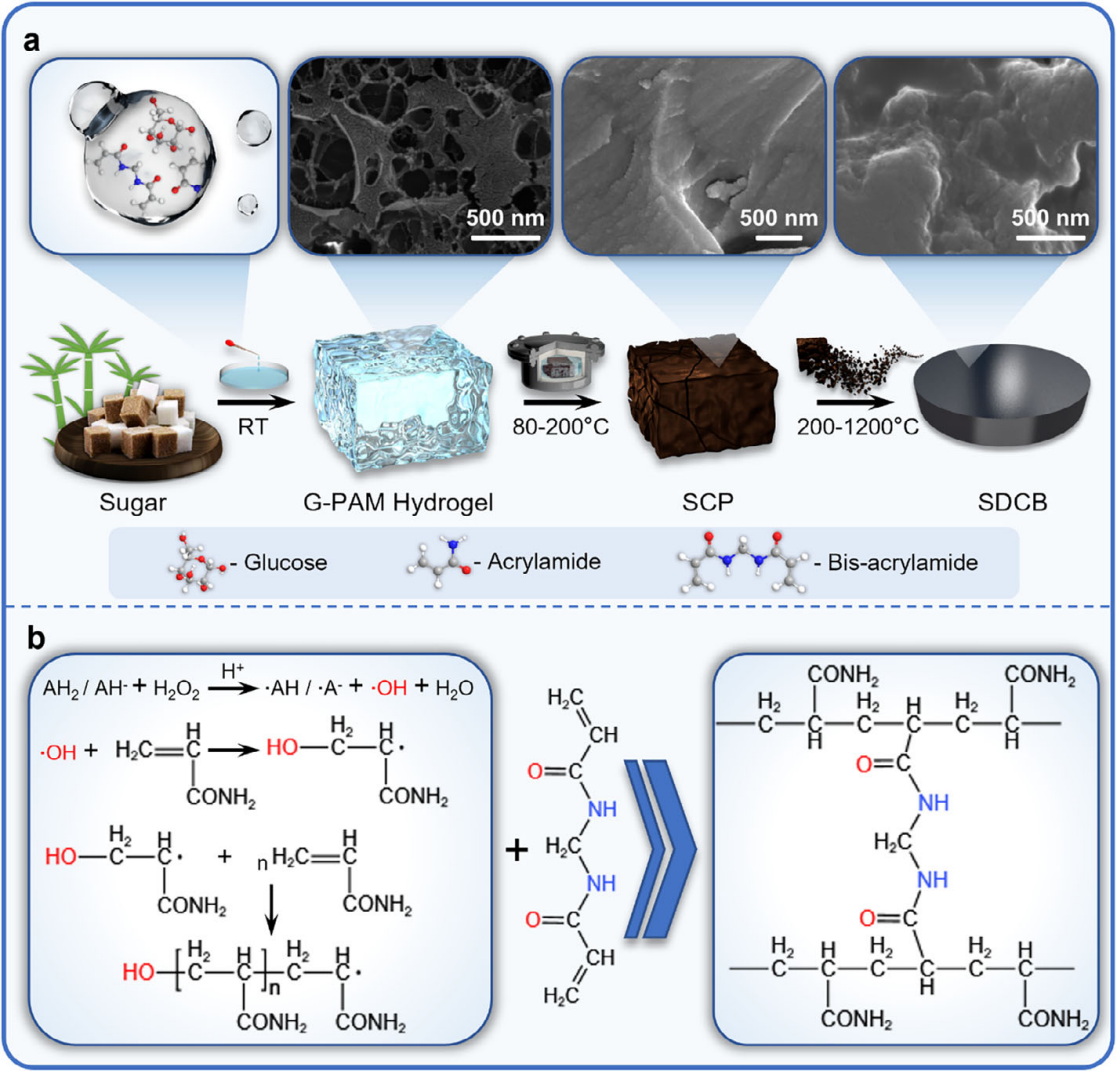
a) Schematic of SHPS strategy. b) The free radical polymerization and crosslinking mechanism of PAM, the gelation process.

Herein, this study managed to obtain a sugar‐derived carbon bulk (SDCB) material through a sugar hydrogel pressureless sintering (SHPS) strategy (**Figure** **1**a). Assisted by the polyacrylamide (PAM) additive, glucose was utilized as the main carbon source and turned into a self‐sintering carbon precursor (SCP) for a more stabilized devolatilization process during sintering. Inspired by the cold sintering theory, a warm compaction process was designed to create compact SCP green bodies.^[^ ^32^, ^33^ ^]^ Contributed by the synergy of the high chemical potential and improved stability of SCP, the pressureless sintering process of sugar‐derived carbon was able to be achieved at a low temperature of 1200 °C. Signs for graphitization were observed in ambient pressure at higher temperatures, confirming the graphitizable nature of SDCBs and distinguishing them from the non‐graphitizable glassy carbon.^[^ ^34^, ^35^, ^36^ ^]^ The EDM‐AF5 (POCO Materials, Entegris) was chosen to be the reference sample since it claims to be the one with the finest average particle size on the market. Investigations regarding their properties, microstructures, and chemical compositions were conducted under the same terms. SDCB samples in the size of *Φ* 37 mm × H 10 mm (Figure S1c, Supporting Information) were successfully produced in the laboratory, suggesting a promising potential for industrial production. The obtained SDCBs displayed a maximum compression strength of 618.8 MPa, a maximum flexural strength of 176.3 MPa, and a bulk density of ≈1.28 g·cm^−3^. Such a combination of high strength and low bulk density has not been reported on bulk carbon materials until now, presenting promising application prospects in the industries of aerospace, precision machining, high‐purity material production, nuclear material, and other high‐temperature fields. The success in low‐temperature pressureless sintering of carbon bulks and the use of sugar‐derived carbon can provide valuable insights for the future sustainable fabrication of high‐performance bulk carbon materials.

## 2 Results and Discussion

Aiming to synthesize densified carbon bulk from sugar, the SHPS strategy was performed in three stages: gelation (RT), dehydration (80–200 °C), and densification (200–1200 °C). The first gelation stage was to solidify the glucose solution and create glucose‐PAM (G‐PAM) hydrogels. The second dehydration stage dehydrates and modifies the G‐PAM hydrogel to turn it into the SCP. It is in the third densification stage that the SDCBs were obtained, who were subsequently subjected to different temperatures and designated as SDCB‐X (X stands for the final heat treatment temperature of the sample).

### 2.1 Synthetic Mechanism

**Figure 2:**
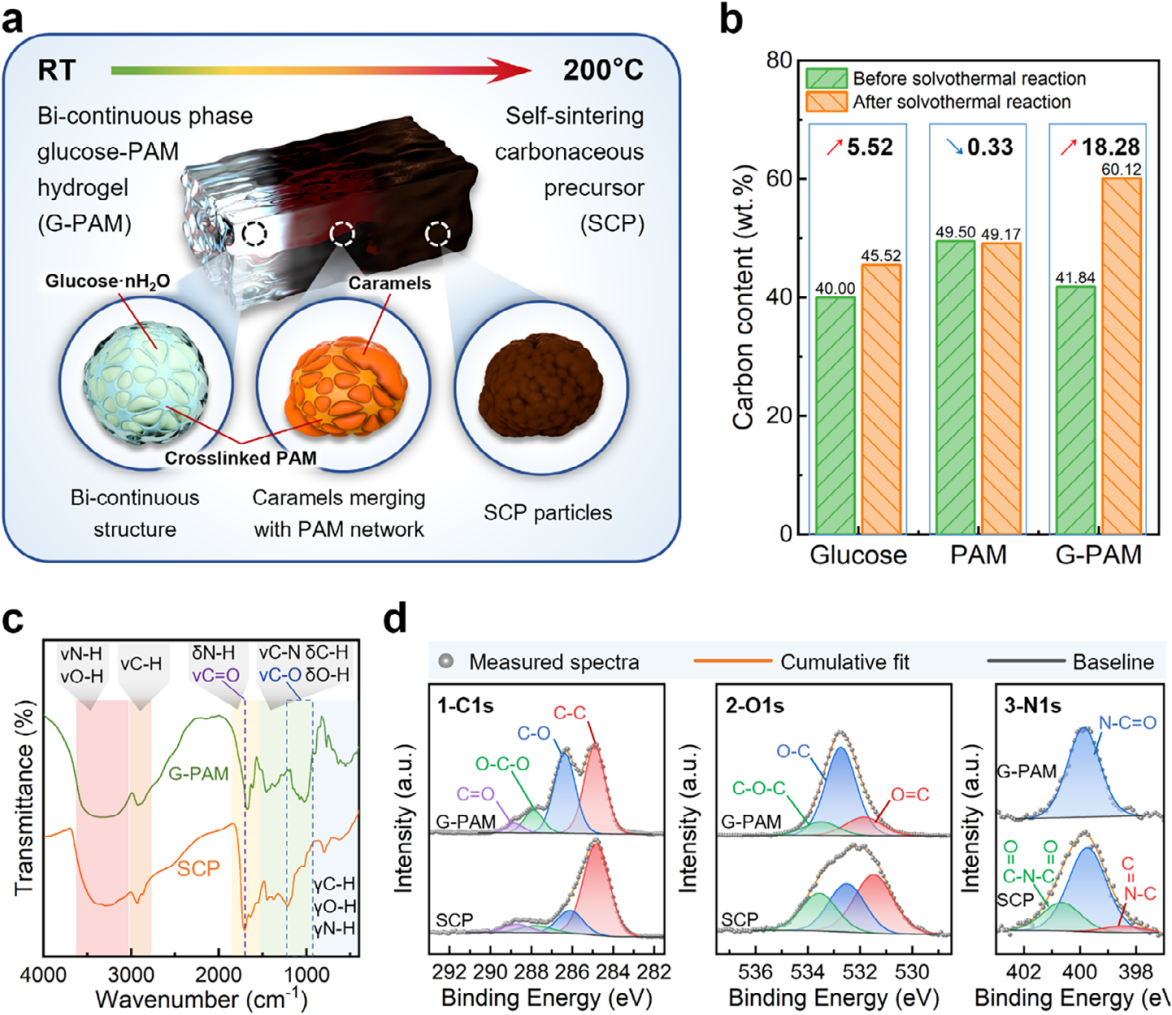
a) Schematic of the dehydration stage. b) Changes in carbon content after the solvothermal dehydration procedure. c) FTIR spectra of G‐PAM and SCP. d) XPS curves of G‐PAM and SCP.

There is a series of organic reactions involved in the gelation and dehydration stages that contribute to the creation of SCP. In the gelation stage, the polymerization and crosslinking among acrylamide (AM) and bis‐acrylamide (bis) molecules was triggered in glucose solution by a redox system composed of hydrogen peroxide (H_2_O_2_) and ascorbic acid (AH_2_).^[^ ^37^ ^]^ As it is shown in Figure 1b, the hydroxyl radicals (·OH) produced from the redox system would attack the AM molecules and initiate chain reactions. With the participation of bis molecules, the PAM chains would be crosslinked to one another and form a 3D skeleton structure growing throughout the glucose solution, which solidifies the solution and turns it into a bi‐continuous phase G‐PAM hydrogel (Figure S1a, Supporting Information). The formation of the hydrophilic PAM network creates extra interfaces for the glucose matrix, which can function as water transportation channels and facilitate the dehydration process. The PAM skeleton also provides structural support for melted glucose. As a result, the uncontrollable foaming behavior of glucose was greatly suppressed (Figure S2, Supporting Information). **Figure** **2**a demonstrates the schematic of structural evolution from G‐PAM to SCP during the dehydration stage, which involves a customized solvothermal procedure that is crucial for the synthesis of SCP. According to Figure 2b (see data source in Figure S3, Supporting Information), such a solvothermal reaction only causes the increase in carbon content in the glucose‐containing samples and poses no significant changes to that of the crosslinked PAM solely. However, the amount of carbon content increase in the G‐PAM (18.28 wt.%) is a lot higher than that of glucose (5.52 wt.%), suggesting that the participation of PAM greatly promotes the dehydration process for glucose. According to the Fourier transform infrared (FTIR) spectra from the G‐PAM to the SCP, the most distinct difference between them is the absence of the multiple νC‐O band at 1200–900 cm^−1^ on the SCP curve, revealing the removal of hydroxyl groups during the solvothermal reaction, which further indicates the disruption of the polyhydroxy aldehyde configuration of glucose. The broad peak that appeared at 1216 cm^−1^ is believed to be attributed to the asymmetric stretching of C─O─C conjugated with the *π*‐bond on furans, considering the overlapped peaks at around 1500 cm^−1^ are signs for unsaturated heterocyclic configurations.^[^ ^38^ ^]^ The intensification of the νC = O peak (1680–1710 cm^−1^) indicates extra carbonyl groups were formed other than those that already existed in amide groups. Such a complex of organic substances with a large amount of unsaturated bonds and rings tends to polymerize among each other and transform into a more stabilized carbon structure during pyrolysis, which contributes to both the carbon conservation and the sintering process. Based on a preliminary analysis of FTIR results, the SCP inherited a complex of molecular structures from caramels, furans, and PAM.^[^ ^38^, ^39^, ^40^, ^41^, ^42^, ^43^, ^44^, ^45^ ^]^ It also revealed that the dehydration behavior in the solvothermal process is mostly a result of dehydroxylation, which is greatly promoted with the participation of PAM. According to the X‐ray photoelectron spectroscopy (XPS) results illustrated in Figure 2d, the C─O peak centered at 286.3 eV in C1s spectra (Figure 2d1) and the O‐C peak centered at 532.5 eV in O1s spectra (Figure 2d2) both decreased sharply from the G‐PAM to the SCP, confirming the removal of hydroxyl groups and occurrence of caramelization. In the N1s spectra (Figure 2d3), the peaks at 400.7 and 398.5 eV corresponding to imide ((C═O)─N─(C═O)) and pyridinium (C─N═C) were able to be separated from the SCP curve, indicating the occurrence of further self‐crosslinking among amide groups and possible formation of aromatic structure. In brief, the obtained SCP is a complex organic substance with a carbon content of 60.12 wt.%, whose conversion from the G‐PAM is the result of caramelization of glucose, self‐crosslinking of PAM, and interactions between them.

### 2.2 Densification Mechanism

**Figure 3:**
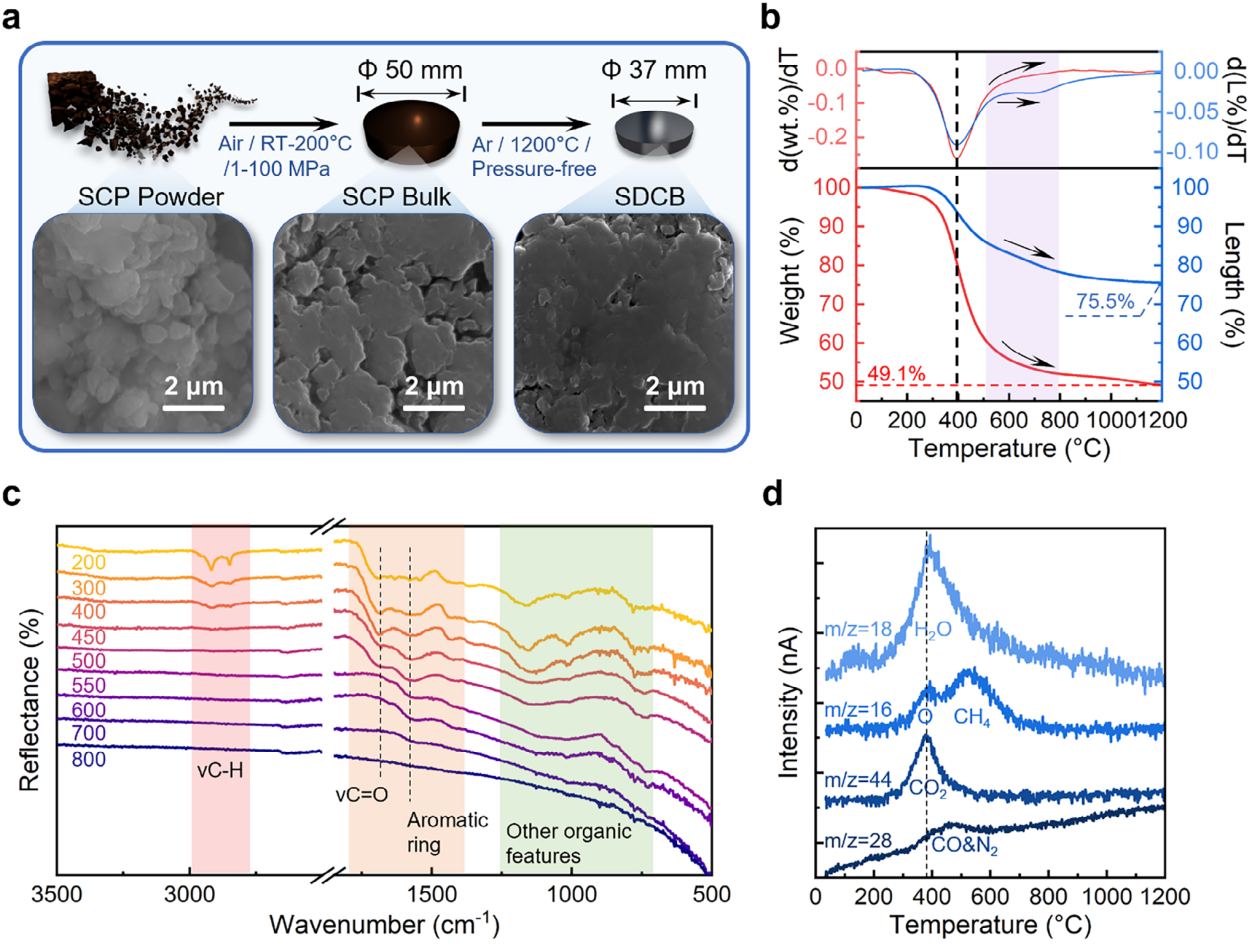
a) Schematic of the densification process. b) TG, thermal expansion, and their first‐order differential curves of the SCP bulk. c) ATR‐FTIR spectra of SCP bulk treated at 200–800 °C. d) MS signals of the tail gas from TG measurement of the SCP bulk.

The densification stage (**Figure** **3**a) consists of two steps: warm compaction and pressureless sintering. The cracked original SCP blocks were pulverized first before they were compacted into densified SCP bulks at 200 °C in air. With the assistance of mild heating, a tight contact among the adjacent SCP particles (Figure 3a, lower center) was formed along with the removal of moisture residue. Then, the SCP bulks were subjected to a pressure‐free heat treatment at 1200 °C in an argon atmosphere and became the SDCBs. Limited by the operating space, intact SCP bulks up to the size of *Φ* 50 mm × H 13 mm (Figure S1b, Supporting Information) were successfully prepared, which would shrink uniformly to the size of ≈*Φ* 37 mm × H 10 mm (Figure S1c, Supporting Information) after pyrolysis at 1200 °C. It is shown in Figure 3a (lower right) that a coherent morphology was formed on the surface of SDCB, revealing the self‐sintering ability of the SCP particles. Considering the simultaneity of sintering behavior and pyrolysis process, the thermal analyses (Figure 3b) regarding the changes of weight (wt.%) and size (L%) were measured in order to investigate the sintering behavior of the SCP bulks. The pyrolysis process of organics always involves the generation of volatiles, which naturally leads to a reduction in weight and a shrinkage in size. The sintering process, on the other hand, usually only causes the shrinkage in size. Therefore, it would be inappropriate to simply regard the shrinkage as a result of sintering while there is a significant decrease in weight. Nevertheless, it is noticed that the decline of the wt.%‐T curve was slowing down in the range of 500–800 °C, while the L%‐T curve seemed to be declining linearly. By comparing their first‐order differential curves, a plateau was spotted on the dL%‐dT curve but not the dwt.%‐dT curve (Figure 3b). It means that, in this particular range of temperature, the weight reduction rate of the sample was slowing down while its size kept shrinking at a constant rate, suggesting a shrinking behavior independent of the mass loss. Such a result allows us to separate the sintering shrinkage from the weight loss shrinkage and confirms the occurrence of sintering behavior. The exothermic peak in the differential scanning calorimetry (DSC) curve (Figure S4a, Supporting Information) of the SCP bulk is located at 403 °C, which matches the maximum weight loss rate point from Figure 3b, confirming the occurrence of the decomposition and devolatilization process. In contrast to the glucose and PAM (Figure S4a, Supporting Information), the exothermic area of the SCP bulk is significantly reduced, suggesting a mitigated decomposition process. Considering the existence of an ongoing sintering behavior, the energy generated from the decomposition reaction is believed to be conveniently absorbed and utilized for the redistribution and diffusion of carbon atoms, which contributes to lowering the sintering temperature.

The attenuated total reflectance FTIR (ATR‐FTIR) spectroscopy (Figure 3c) was utilized to characterize the transformation of the SCP bulk from 200 to 800 °C. As the treatment temperature increased, it was observed that the peaks at around 2915 and 2845 cm^−1^ attributed to νC‐H gradually disappeared after 450 °C, indicating the degradation of the organic carbon chain. A similar situation also happened to the νC = O peak at around 1700 cm^−1^ and the νC‐O peaks at around 1160 and 1005 cm^−1^, only they disappeared after 500 °C, which means other organic functional groups were degrading simultaneously. The aromatic ring bands at ≈1560 and 1430 cm^−1^ were found growing with the temperature before 500 °C, and then started to diminish and finally disappeared after 700 °C. Such patterns suggest that the SCP bulks started their degradation at ≈300 °C, including the removal of oxygen‐containing functional groups, breaking of long carbon chains, and aromatization, which came to an end at 500 °C and began the ultimate conversion toward a pure carbon structure. The early degradation of SCP at 300 °C reveals its relatively high chemical potential, which significantly reduces the activation energy required for sintering and helps to lower the sintering temperature.

A mass spectrometry (MS) device was attached to the exhaust end of the Thermogravimetry (TG) analyzer to identify the composition of the volatiles produced during the pyrolysis of SCP bulks. According to the results (Figure 3d), typical signals of m/z = 16 (CH_4_), 18 (H_2_O), 28 (N_2_ & CO), and 44 (CO_2_) were detected mostly within the range of 300–800 °C, which matches the massive weight loss area in Figure 3b. The signals of H_2_O and CO_2_ reached their peak at around 400 °C, which unsurprisingly matches the maximum weight loss rate point. The H_2_O signal displayed the strongest intensity, indicating a preferential elimination of hydrogen and oxygen content, which favors the goal of carbon conservation. With the removal of oxygen‐containing groups, the redundant hydrogen starts to escape with carbon in the form of hydrocarbons (mostly CH_4_), hence the m/z = 16 peak at the range of 400–700 °C. It is reassuring to find out that the nitrogen mostly escaped in the form of N_2_, for it is harmless to the atmosphere. More detailed MS results regarding the exhaust composition can be found in Figure S5 (Supporting Information), and most of the detections are friendly to the environment. In short, the sintering behavior takes place simultaneously with the pyrolytic transformation (Figure S6, Supporting Information) from SCP bulk to SDCB, which involves a complex of decomposition reactions that produce H_2_O, CO_2_, CH_4_, N_2_, CO, etc. The whole process is able to be completed within 1200 °C and poses minimum damage to the environment.

### 2.3 Properties and Microstructure

**Figure 4:**
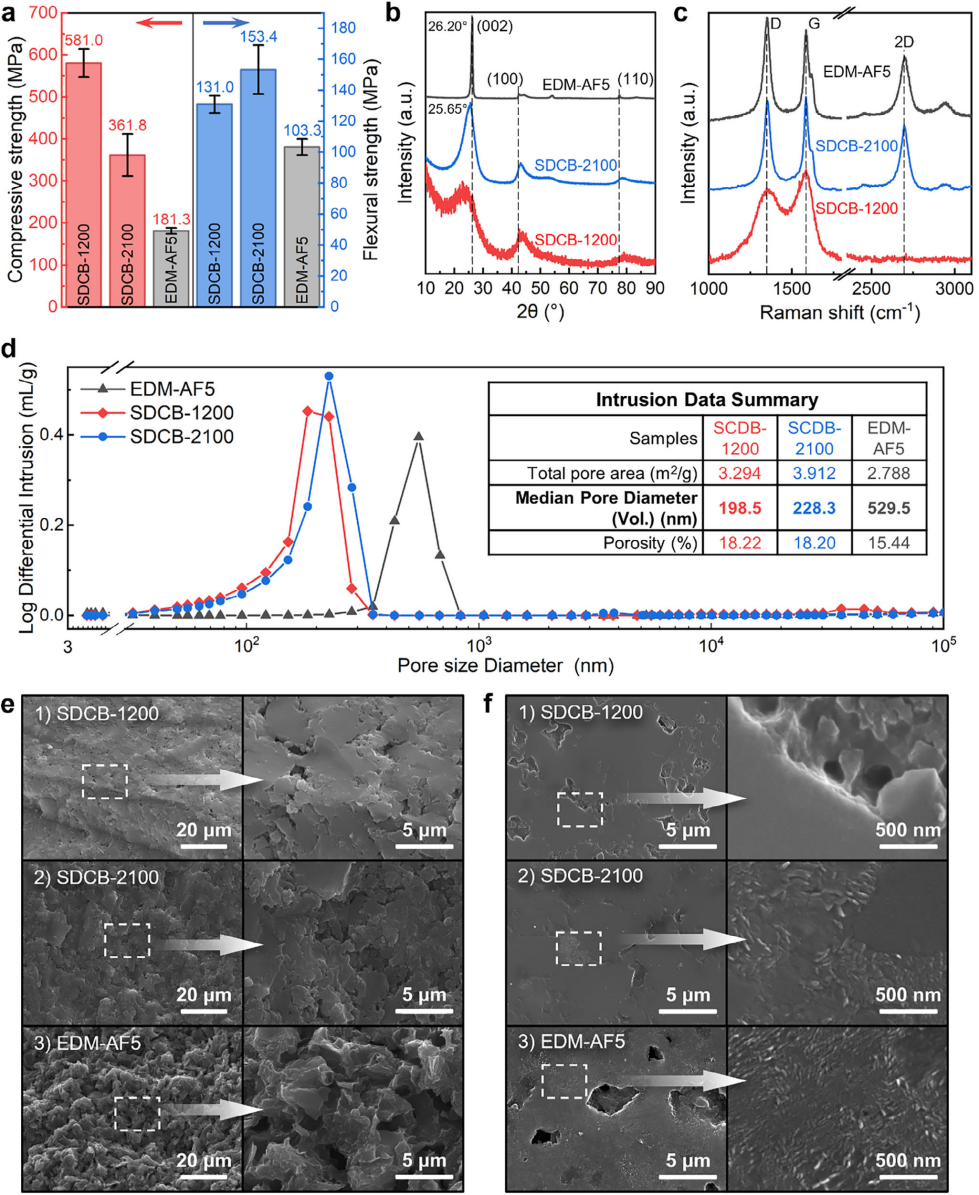
a) Mechanical properties. b) Raman spectra. c) XRD patterns. d) MIP curves. e) SEM images of fracture surface. f) SEM images of polished surface.

EDM‐AF5 (POCO Materials, Entegris), a top‐grade graphite product with an average particle size less than 1 µm, was purchased as the reference sample. The carbon contents of the fabricated SDCB samples, along with the reference sample, were measured and illustrated in Figure S3 (Supporting Information). The carbon content of the SDCB had already reached 96.5 wt.% at 1200 °C, which is close to the 97.6 wt.% of EDM‐AF5, and an even higher carbon content of 99.1 wt.% was able to be obtained at 2100 °C, showing great potential in achieving ultra‐high purity. Tests of mechanical properties (Table S2 and Figure S7, Supporting Information) were carried out in order to determine the strength of the SDCBs, and the same terms were applied to the reference sample. According to the measured data presented in **Figure** **4**a, the SDCB‐1200 showed an exceedingly high average compressive strength of 581.0 MPa, and that of the SDCB‐2100 was 361.8 MPa, both of which are a lot higher than the 181.3 MPa of the EDM‐AF5. Differently, the average flexural strength actually increased from the 131.0 MPa of SDCB‐1200 to the 153.4 MPa of SDCB‐2100, both obviously higher than the 103.3 MPa of the EDM‐AF5. With such a high strength, the SDCBs displayed a low bulk density of ≈1.28 g cm^−3^ (Table S2, Supporting Information), much lower than the 1.769 g cm^−3^ of EDM‐AF5. Although they share a similar bulk density, the skeleton density decreased by 15% from SDCB‐1200 to SDCB‐2100, causing a rise in relative density, which was also displayed visually in Figure 4e,f. While the higher flexural strength of SDCB‐2100 can be explained by the increase of relative density, the decrease of compressive strength is believed to be a result of graphitization. X‐ray diffraction (XRD) was utilized to investigate the crystal structure of the SDCBs. According to the results displayed in Figure 4b, a distinct peak near 26° corresponds to the (002) plane of the graphite crystal is observed in SDCB‐2100, revealing its graphitic carbon structure. The SDCB‐1200 displayed a typical XRD pattern of an amorphous carbon structure as expected. Similar results were also observed in their Raman spectra, illustrated in Figure 4c. The overlapping between the D band (≈1350 cm^−1^) and G band (≈1580 cm^−1^) of the SCDB‐1200 indicates an amorphous carbon structure, and the absence of the 2D (≈2700 cm^−1^) confirms the lack of graphite structure. Yet, the sharp and distinct D, G, and 2D peaks were observed in the SCDB‐2100 spectrum, revealing the existence of a graphite structure. The spectrum feature of the SCDB‐2100 was found to be similar to that of the EDM‐AF5, which had been through a complete graphitization procedure. The D band is attributed to a breathing mode of sixfold carbon rings, which requires defects for its activation in the graphite structure.^[^ ^46^, ^47^ ^]^ The G band is corresponding to the in‐plane stretching motion of pairs of carbon *sp^2^* atoms, representing the graphite structure.^[^ ^46^, ^47^ ^]^ Therefore, the intensity ratio of *I_D_/I_G_* is commonly used to describe the state of graphitization.^[^ ^46^, ^47^, ^48^ ^]^ The *I_D_/I_G_* value of the SDCB‐2100 curve is 0.96, less than the 1.13 of the EDM‐AF5, indicating a less defected graphitic carbon structure than the EDM‐AF5. The 2D peak is the overtone of the D peak, which is sensitive to the interlayer coupling of graphene layers and doesn't require defects for its activation.^[^ ^49^, ^50^, ^51^, ^52^ ^]^ As a result, the shape of the 2D peak is related to the stacking status of graphene layers. Both of the 2D peaks in the Raman spectra of the SDCB‐2100 and EDM‐AF5 were symmetric and only one Lorentzian profile (Figure S8, Supporting Information) was required for their peak fitting, showing a similar feature to single layer graphene. However, the full width at half maximum (FWHM) of the 2D peaks in the EDM‐AF5 and SDCB‐2100 are 67.5 and 59.4 cm^−1^, which are broader than the 30 cm^−1^ usually seen in single‐layer graphene.^[^ ^53^ ^]^ Plus the high intensity of D peaks, their Raman spectrum fit the description of turbostratic graphite.^[^ ^49^ ^]^ According to the XRD data in Figure 4b, the calculated (002) interplanar distance (*d_002_*) of SDCB‐2100 is 0.347 nm, larger than the 0.335 nm of perfect graphite, which explains the symmetry of its 2D peak. Although the EDM‐AF5 also displayed a large *d_002_* of 0.340 nm, the narrow and sharp shape of its (002) peak indicates the presence of highly ordered graphite crystals. Moreover, according to the Scherrer equation, the average crystallite size (*L_a_*) of SDCB‐2100 is calculated to be 8.14 nm, suggesting a nanoscale polycrystalline graphitic carbon structure. It is reasonable for EDM‐AF5 to present a larger *L_a_* of 14.86 nm, considering the presence of coarse graphite crystal. Thus, the finer graphitic structure of SDCB‐2100 is believed to be one of the factors that contributes to its high strength. In order to deal with the sintering challenge of bulk carbon material, the products from conventional industry are inevitably left with pores, which directly affect their mechanical performances. According to the mercury intrusion porosimetry (MIP) data illustrated in Figure 4d, the SDCBs presented a pore distribution centered at ≈200 nm, which is smaller than half that of the EDM‐AF5. Such a finer pore structure is another factor that contributes to the high strengths and low densities of SDCBs.

Scanning electron microscopy (SEM) was utilized in Figure 4e to illustrate the fracture surfaces of the samples broken in the mechanical properties test. The exfoliating morphology observed on the EDM‐AF5 fracture surface (Figure 4e3) indicates an intergranular fracture behavior, which exposes its coarse grains with poor interfacial bonding that weakens the mechanical strength. Yet, SDCBs displayed a transgranular dominant fracture feature of a flat and even morphology (Figure 4e1,4e2), which reveals their strong interfacial bonding. Their coherent and uniform morphology (Figure 4f1,4f2) visually demonstrated a well‐sintered microstructure. Moreover, the nanoscale polycrystalline graphite structures exposed on the polished surface of EDM‐AF5 (Figure 4f3) were also observed in SDCB‐2100 (Figure 4f2), which confirms its graphitization behavior. In addition, it should be noted that the pore sizes exposed on their polished surfaces match their MIP results.

**Figure 5:**
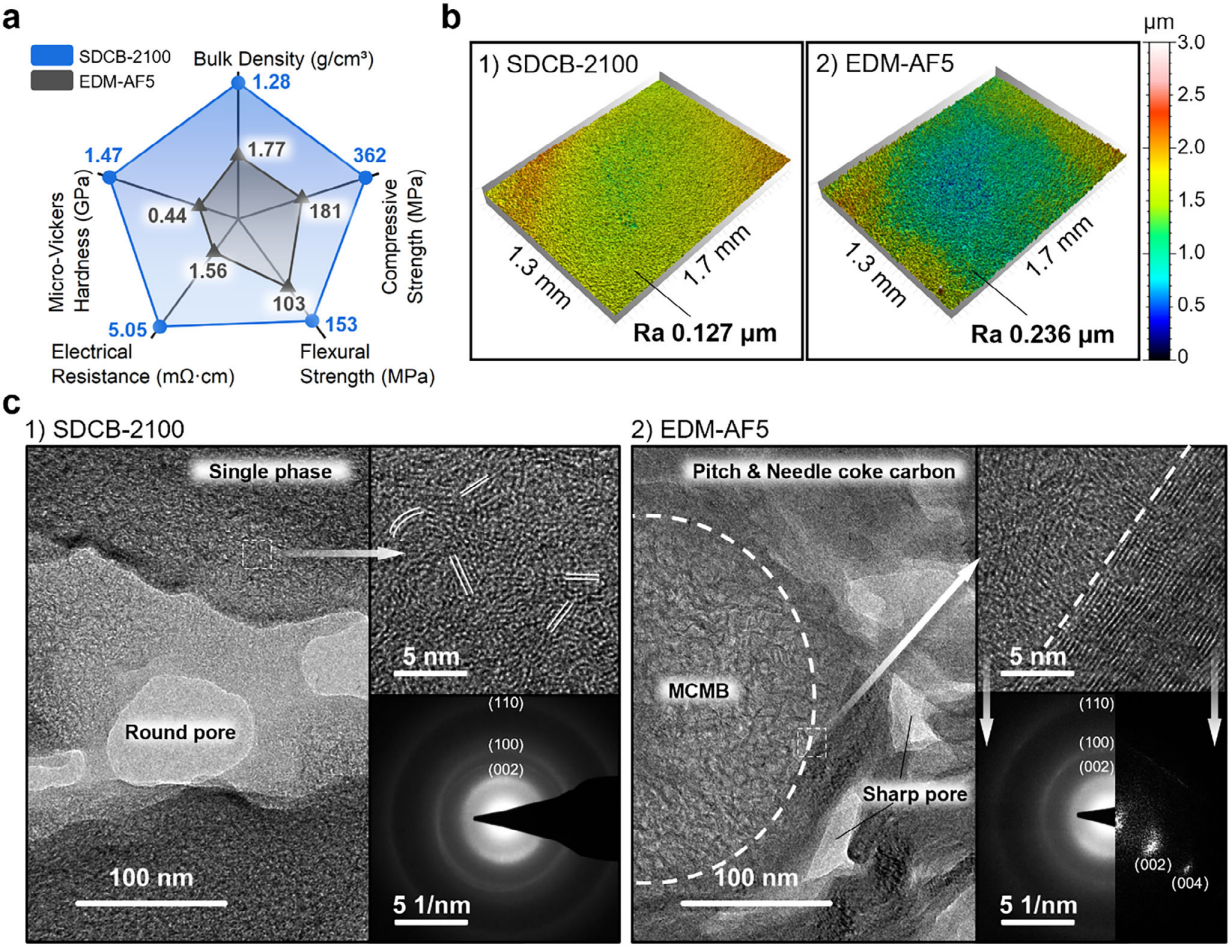
a) Comparison of properties. b) Polished surface morphology. c) Microstructure.

In the field of high precision EDM electrode material, better surface quality and higher electrical resistance means higher precision.^[^ ^8^, ^9^, ^10^, ^11^ ^]^ **Figure** **5**a compares the SDCB‐2100 with the EDM‐AF5 in five different properties, and the EDM‐AF5 was outmatched in every one of them. As shown in Figure 5b, after the exact same polishing procedure, the SDCB‐2100 exhibited a surface roughness (Ra) of 0.127 µm, much lower than the 0.236 µm of EDM‐AF5. Moreover, their C1s spectra (Figure S9, Supporting Information) reveal that they have similar *sp^3^/sp^2^* compositions, and it is believed that the presence of *sp^3^* carbon benefits their mechanical performances.^[^ ^17^, ^19^ ^]^ In short, the SDCB‐2100 is a lot stronger and lighter than the EDM‐AF5, and it is easier for the SDCB‐2100 to acquire better surface quality.

Transmission electron microscopy (TEM) (Figure 5c) was utilized to characterize the microstructure of the SDCB‐2100 and the EDM‐AF5 at the nanoscale. The SDCB‐2100 presented a uniform carbon structure consisting of randomly stacked graphite layers (Figure 5c1, upper right), agreeing with its XRD and Raman patterns. The EDM‐AF5 displayed a distinct two‐phase carbon structure (Figure 5c2, upper right), the one with the turbostratic graphite structure in the spherical area is believed to be formed from the MCMB, and the other with the highly ordered stacking graphite layers is more likely to be formed from needle coke or mesophase pitch. Such a two‐phase structure matches well with its XRD and Raman patterns. Sintering necks and round pores were observed in the TEM image of SDCB‐2100 (Figure 5c1), which also contributes to its high strength by resisting stress concentration. The sharp concentric ring pattern observed in the selected‐area electron diffraction (SAED) (Figure 5c1, lower right) of the SDCB‐2100 confirms its randomly oriented nano‐polycrystalline microstructure. Furthermore, the presence of multiple defects along the boundary between the two phases of EDM‐AF5 confirms our concerns about the poor interfacial bonding of conventional bulk carbon materials.

**Figure 6:**
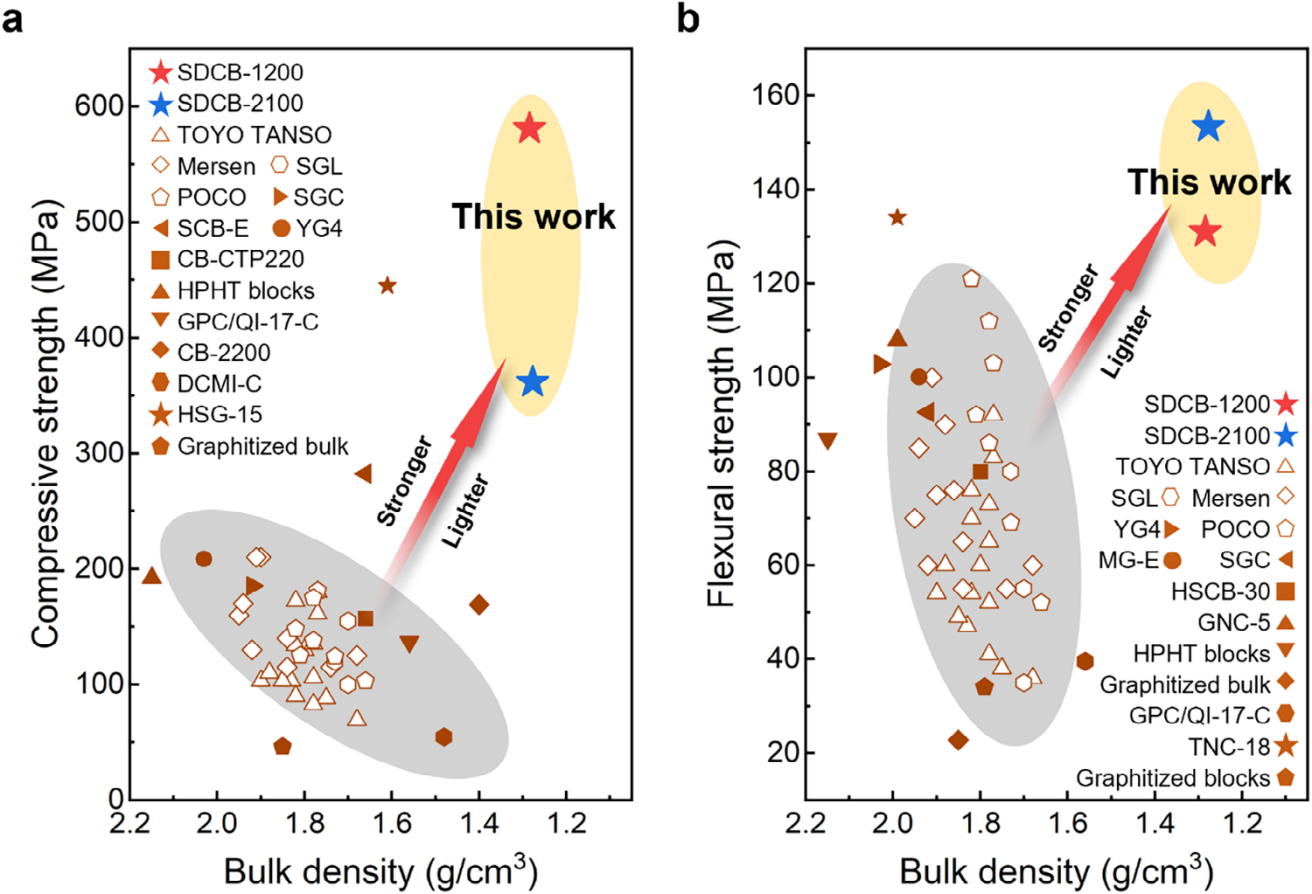
Comparison of mechanical strengths with respect to density. (The detailed data can be found in Tables S1 and S2, Supporting Information.).

The flexural strength and compressive strength versus bulk densities of bulk carbon materials from different sources were collected and illustrated in **Figure** **6**, presenting advantages of SDCBs in terms of both strength and lightweightness. It is worth mentioning that the graphitizable nature of SDCBs is confirmed by their graphitic features (Figure S10, Supporting Information) after being treated at 3000 °C under ambient pressure. Additionally, the universality of SHPS for different sugars were validated by fabricating SDCBs using sucrose and fructose (Figure S11, Supporting Information).

## 3 Conclusion

In this study, a high‐strength yet lightweight and graphitizable bulk carbon material (SDCB) has been sintered pressurelessly at a low temperature of 1200 °C using a precursor (SCP) synthesized from glucose. The sintering process was significantly promoted by the entropy increase and exothermic effects from the decomposition of SCP, while the activation energy required for sintering was also reduced due to the high chemical potential of SCP. The obtained SDCBs displayed a superior mechanical performance (average compressive strength of 581.0 MPa, average flexural strength of 153.4 MPa) with a low bulk density (≈ 1.28 g cm^−3^), thanks to its uniform microstructure composed of interlocked nanocrystalline graphite, amorphous carbon, and near‐spherical pores. Moreover, the size of the artifact was able to reach *Φ* 37 mm × H 10 mm in the laboratory stage, exhibiting a promising potential for scalable production. The not fully densified structure of SDCBs suggests that there is still a lot of room for further enhancement. Bulk carbon materials with such advanced properties present promising application prospects in the fields of spacecraft thermal protection, precision machining, and the nuclear industry.

## 4 Experimental Section

### Reagents

Glucose monohydrate (AR) and sucrose (AR) were purchased from Tianjin ZhiYuan Reagent Co., Ltd. D‐fructose (99.0%) was purchased from Beijing Biotopped Technology Co., Ltd. Acrylamide (98.0%) was purchased from Tianjin Kermel Chemical Reagent Co., Ltd. Bis‐acrylamide (99%) was purchased from Shanghai Aladdin Bio‐Chem Technology Co., Ltd. Hydrogen peroxide solution (30%, H_2_O_2_) was purchased from Tianli Chemical Reagent Co., Ltd. L‐Ascorbic acid (99.7%) was purchased from Tianjin Municipality Tianxin Superfine Chemical Industry Development Center. Acetone (99.5%) was purchased from Tianjin Fuyu Fine Chemical Co., Ltd. All chemical reagents were used without further purification.

### Preparation of the G‐PAM Hydrogel Samples

Bis‐acrylamide (0.9 g), glucose·H_2_O (198 g), acrylamide (36 g), were dissolved in pure water (72 g) sequentially at 60 °C within 45 min. After it had cooled down below 40 °C, 20 drops (≈1 g) of ascorbic acid solution (5 wt.%) and H_2_O_2_ solution (2 wt.%) were added into the solution while stirring. Then the mixed solution was poured into rubber molds and put still for gelation, after which the GPAM hydrogel was obtained.

### Preparation of the SCP Samples

The G‐PAM hydrogels were first dried at 80 °C for 24–72 h before they were subjected to a solvothermal process in a sealed reactor half‐filled with acetone at 200 °C for 4–10 h (depending on the amount) and became SCP blocks, which were dried at 100 °C for at least 2 h before they were bagged and sealed for use.

### Preparation of the SDCB Samples

The SCP blocks were shattered first, and the particles under the size of 1 mm were sieved out for further grinding using planetary ball milling (360 r min^−1^ for 4 h). The acquired SCP powders were compacted into SCP bulks at 200 °C, which were subsequently treated at 1200 °C for 2 h in an argon atmosphere and became the SDCB‐1200. The SDCB‐2100 samples were fabricated by subjecting SDCB‐1200 to a graphitization process at 2100 °C for 1 h. Particularly, the SDCB‐3000 were fabricated by subjecting SDCB‐1200 to a graphitization process at 3000 °C for 10 h. The heating rate of the pyrolysis and graphitization was controlled below 5 °C min^−1^.

### Chemical Composition

Measured by an organic elemental analyzer (Vario EL cube, Elementar).

### FTIR & ATR‐FTIR

Measured by a Fourier transform infrared spectrometer (Nicolet iS50). The spectra were recorded in the range of 4000–400 cm^−1^ with a wavenumber accuracy of 0.005 cm^−1^. Additionally, the samples tested in Figure 3c were prepared by subjecting SCP tablets to the target temperatures (200–800 °C, 5 °C min^−1^) for 10 min in an argon atmosphere.

### X‐ray photoelectron spectroscopy (XPS)

Conducted on a Thermo ScientificTM ESCALAB 250Xi spectrometer equipped with a monochromatic Al Kα X‐ray source (1486.6 eV) operating at 150 W. Samples were analyzed under vacuum (*p* < 10–8 mbar) with a pass energy of 150 eV (survey scans) or 30 eV (high‐resolution scans).

### X‐ray Diffraction Analysis (XRD)

Measured by an X‐ray diffractometer (Empyrean, Panalytical) with a copper target (Kα, λ = 1.5406 Å). A step size of 0.05° and a time interval of 0.5 s were used.

### Raman spectroscopy

Measured by a tip‐enhanced laser confocal Raman spectroscopy system (inVia‐Reflex, Renishaw) with a laser wavelength of 532 nm.

### Thermal analyses

The sintering and pyrolysis process from the SCP bulk to the SDCB were studied through thermogravimetry (TG) and differential scanning calorimetry (DSC) in argon atmosphere using the NETZSCH STA 449 F3 Jupiter system. The analysis was conducted from room temperature to 1200 °C with a heating rate of 5 °C min^−1^, during which the produced exhaust was characterized by a mass spectrometer accessory (QMS 403 D‐IS 50). The shrinking behavior from the SCP bulk to the SDCB was characterized by a thermal dilatometer (402 Expedis Supreme) with the same heating parameters as the TG analysis, and the samples were prepared in the size of 4 × 4 × 25 mm.

### Flexural and compression strength

Measured by a universal testing machine (Instron 5569, USA). The three‐point flexural test samples were prepared in the size of 3 × 4 × 22 mm and tested with a loading rate of 0.5 mm min^−1^ and a span of 16 mm. The compression test samples were prepared in the size of 4 × 4 × 8 mm and tested under a loading rate of 0.5 mm min^−1^.

The formula for flexural strength (𝜎_f_):

(1) $$\sigma_{f}=\frac{3P_{f}L}{2bh^{2}}$$

𝜎_f_ — Flexural strength (MPa);

𝑃_f_ — maximum load (N);

𝐿 — span length (mm);

ℎ — sample height (mm);

𝑏 — sample width (mm).

The formula for compressive strength (σ_c_):

(2) $$\sigma_{c}=\frac{P}{A}$$

𝜎_𝑐_ — compressive strength (MPa);

𝑃 — applied load (N);

𝐴 — load‐bearing area (mm^2^).

### Micro‐Vickers hardness

Measured by a micro‐Vickers indenter (Wilson Tukon1102) with a load of 0.98 N and a dwell time of 10 s. The testing surfaces of the samples were subjected to a P240‐P480‐P1000‐P2000 graded grinding procedure, then a rough polishing procedure with 3.5 µm diamond suspension, and at last a fine polishing procedure with 1 µm diamond suspension.

### True density (skeleton density)

Measured by a fully automatic gas displacement true density meter (AccuPyc II 1340).

### Mercury intrusion porosimetry (MIP)

Measured by an automatic mercury porosimeter (AutoPore lv9620, Micromeritics).

### 3D surface profile

Measured by a 3D profiler (ContourGT‐X). The testing surfaces of the samples were all subjected to a polishing procedure exactly the same as the one performed in the micro‐Vickers hardness measurement.

### Electrical resistance

Measured by a multifunctional digital four‐probe meter (ST2258C) using ST2558B‐F01 probe.

### Scanning electron microscopy (SEM)

Most of the carbonized samples were observed using a scanning electron microscope (TESCAN CLARA, Zeiss Merlin Compact) at an accelerating voltage of 20 kV. Some organic samples were observed at an accelerating voltage of 2–5 kV. The microstructure of the freshly made hydrogel samples was observed using a cryo‐scanning electron microscope (Cryo‐SEM, Regulus8220) at a cold stage temperature of −140 °C and an accelerating voltage of 5 kV.

### Transmission electron microscopy (TEM)

Focused ion beam (FIB) milling (TESCAN CLARA) was used to extract and thin micro‐scale samples to ≈50 nm, which were then transferred to TEM grids. During FIB processing, the voltage and current were gradually reduced from 20 kV and 0.6–1 nA to 5 kV and 20–30 pA. Final polishing was performed at 5 kV and 10 pA. The microstructures of the recovered samples were characterized by a monochromated, aberration‐corrected Titan G2 60–300 FEI operating at an accelerating voltage of 300 kV.

## Conflict of Interest

The authors declare no conflict of interest.

## Author Contributions

C.H., D.C., and B.S. conceived the idea and designed the experiments. X.Z. supervised the research. C.H. performed the experiments and analysed the data. M.T., R.P., and W.Z. helped to perform experiments. G.C. and W.B. helped with microstructure characterization. C.H. and M.T. co‐wrote the manuscript. S.D. and J.H. helped with the spectra characterization. All authors discussed the results. All authors participated in discussions of the research.

## Supporting information



Supporting Information

## Data Availability

The data that support the findings of this study are available from the corresponding author upon reasonable request.

## References

[1] H. E. Martens , W. V. Kotlensky , J. Mater. Process. Technol. 1960, 186, 960.

[2] C. Karthik , J. Kane , D. P. Butt , W. E. Windes , R. Ubic , Microsc. Microanal. 2012, 18, 272. 22264445 10.1017/S1431927611012360

[3] M. Inagaki , F. Kang , in Mater. Sci. Eng. Carbon Fundam. Second, (Eds: M. Inagaki , F. Kang ), Butterworth‐Heinemann, Oxford, 2014, pp. 219–525.

[4] Z. He , J. Song , Z. Wang , X. Guo , Z. Liu , T. J. Marrow , Fuel 2021, 290, 120055.

[5] C. Karthik , J. Kane , D. P. Butt , W. E. Windes , R. Ubic , J. Adv. Ceram. 2022, 196, 474.

[6] T. Czelusniak , C. F. Higa , R. D. Torres , C. A. H. Laurindo , J. M. F. de Paiva Júnior , A. Lohrengel , F. L. Amorim , J. Braz. Soc. Mech. Sci. Eng. 2019, 41, 14.

[7] M. S. Sisodiya , P. Agarwal , Eng. Res. Express 2024, 6, 012506.

[8] A. Muttamara , Y. Fukuzawa , N. Mohri , T. Tani , J. Mater. Process. Technol. 2009, 209, 2545.

[9] A. Suresh , S. J. Rowan , C. Liu , H. E. MARTENS , W. V. Kotlensky , K. L. Aas , Microsc. Microanal. 2024, 13, 1453.

[10] T. Koyano , Y. Sugata , A. Hosokawa , T. Furumoto , Precis. Eng 2019, 55, 95.

[11] T. Koyano , Y. Sugata , A. Hosokawa , T. Furumoto , Precis. Eng. 2017, 47, 480.

[12] H. Zhao , Z. He , X. Guo , P. Lian , Z. Liu , New Carbon Mater 2020, 35, 184.

[13] D. K. Sam , H. Li , Y.‐T. Xu , Y. Cao , New Carbon Mater 2017, 159, 369.

[14] K. Shen , Z.‐H. Huang , J. Yang , W. Shen , F. Kang , Carbon 2011, 49, 3200.

[15] H. Jander , C. Borchers , H. Böhm , A. Emelianov , C. Schulz , Carbon 2019, 150, 244.

[16] R. Tan , Z. Fan , Z. Xie , M. Zhang , K. He , Q. Huang , Carbon 2016, 101, 439.

[17] B. Miao , J. Wang , J. Li , S. Gu , L. Wang , W. Jiang , J. Adv. Ceram. 2022, 11, 1815.

[18] J. Ran , K. Lin , H. Yang , J. Li , L. Wang , W. Jiang , Appl. Phys. A 2018, 124, 262.

[19] K. Lin , H. Fang , A. Gao , H. Yu , L. Wang , Q. Yu , L. Gu , Q. Zhang , J. Li , W. Jiang , Adv. Mater. 2021, 243, 2007513. 10.1002/adma.202007513 33738845

[20] H. He , R. Zhang , P. Zhang , P. Wang , N. Chen , B. Qian , L. Zhang , J. Yu , B. Dai , Adv. Sci. 2023, 10, 2205557. 10.1002/advs.202205557 PMC10238227 36988448

[21] K. Zhang , Z. Huang , M. Yang , M. Liu , Y. Zhou , J. Zhan , Y. Zhou , Sus. Mat. 2023, 3, 558.

[22] S. Wu , D. Chen , G. Zhao , Y. Cheng , B. Sun , X. Yan , W. Han , G. Chen , X. Zhang , Chem. Eng. J. 2022, 434, 134514.

[23] Y. Yang , D. Chen , Y. Cheng , B. Sun , G. Zhao , W. Fei , W. Han , J. Han , X. Zhang , Green Chem. 2022, 24, 5097.

[24] M. Tan , D. Chen , Y. Cheng , H. Sun , G. Chen , S. Dong , G. Zhao , B. Sun , S. Wu , W. Zhang , J. Han , W. Han , X. Zhang , Adv. Funct. Mater. 2022, 32, 2202057.

[25] H. Qu , X. Zhang , J. Zhan , W. Sun , Z. Si , H. Chen , ACS Sustain. Chem. Eng. 2018, 6, 7380.

[26] S. Pak , J. Ahn , H. Kim , Carbohydr. Polym. 2022, 296, 119948. 36088028 10.1016/j.carbpol.2022.119948

[27] X. Wang , Y. Zhang , C. Zhi , X. Wang , D. Tang , Y. Xu , Q. Weng , X. Jiang , M. Mitome , D. Golberg , Y. Bando , Nat. Commun. 2013, 4, 2905. 24336225 10.1038/ncomms3905 PMC3905699

[28] S. Zhang , S.‐F. Jiang , B.‐C. Huang , X.‐C. Shen , W.‐J. Chen , T.‐P. Zhou , H.‐Y. Cheng , B.‐H. Cheng , C.‐Z. Wu , W.‐W. Li , H. Jiang , H.‐Q. Yu , Nat. Sustain. 2020, 3, 753.

[29] J. Hao , F. Xu , D. Yang , B. Wang , Y. Qiao , Y. Tian , Renew. Sustain. Energy Rev. 2025, 208, 115090.

[30] B. Sun , D. Chen , Y. Cheng , W. Fei , D. Jiang , S. Tang , G. Zhao , J. Song , C. Hou , W. Zhang , S. Wu , Y. Yang , M. Tan , J. Zhang , D. Wei , C. Guo , W. Zhang , S. Dong , S. Du , J. Han , J. Luo , X. Zhang , Adv. Mater. 2022, 34, 2200363. 10.1002/adma.202200363 35686916

[31] W. Zhang , M. Tan , D. Chen , B. Sun , Y. Cheng , L. Xun , J. Hu , C. Hou , Y. Liu , Y. Fang , P. Hu , W. Han , S. Dong , S. Du , J. Han , S. Miao , Q. Yang , Y. Zhou , X. Zhang , Adv. Mater. 2024, 36, 2309899. 10.1002/adma.202309899 37884393

[32] C. Tu , L. Hong , T. Song , X. Li , Q. Dou , Y. Ding , T. Liao , S. Zhang , G. Gao , Z. Wang , Y. Jiang , X. Li , L. Li , M. Si , X. Tai , H. Wang , J. Guo , J. Adv. Ceram. 2024, 13, 1453.

[33] A. Muttamara , Y. Fukuzawa , N. Mohri , T. Tani , J. Food Sci. 2024, 13, 1697.

[34] K. J. Putman , M. V. Sofianos , M. R. Rowles , P. J. F. Harris , C. E. Buckley , N. A. Marks , I. Suarez‐Martinez , Carbon 2018, 135, 157.

[35] X. Dou , I. Hasa , D. Saurel , C. Vaalma , L. Wu , D. Buchholz , D. Bresser , S. Komaba , S. Passerini , Mater. Today 2019, 23, 87.

[36] R. E. Franklin , Math. Phys. Eng. Sci. 1951, 209, 196.

[37] L. Xu , Y. Tong , G. Wu , J. Beijing Inst. Chem. Technol. 1994, 208, 27.

[38] Y. Sun , K. Sun , L. Zhang , S. Zhang , Q. Liu , Y. Wang , T. Wei , G. Gao , X. Hu , Energy Fuels 2020, 34, 3250.

[39] M. Tutaş , M. Sağlam , M. Yüksel , J. Anal. Appl. Pyrolysis 1991, 22, 129.

[40] A. Golon , N. Kuhnert , J. Agric. Food Chem. 2012, 60, 3266. 22375847 10.1021/jf204807z

[41] M. del , P. Buera , J. Chirife , S. L. Resnik , R. D. Lozano , J. Food Sci. 1987, 52, 1059.

[42] A. Muttamara , Y. Fukuzawa , N. Mohri , T. Tani , J. Food Sci. 1966, 31, 561.

[43] A. H. Motagamwala , K. Huang , C. T. Maravelias , J. A. Dumesic , Energy Environ. Sci. 2019, 12, 2212.

[44] S. M. Süren , R. Tutar , C. Özeroğlu , S. Karakuş , J. Polym. Environ. 2024, 32, 164.

[45] K. Ou , X. Dong , C. Qin , X. Ji , J. He , Mater. Sci. Eng. C 2017, 77, 1017. 10.1016/j.msec.2017.03.287 28531973

[46] A. C. Ferrari , J. Robertson , Phys. Rev. B 2000, 61, 14095.

[47] A. C. Ferrari , D. M. Basko , Nat. Nanotechnol. 2013, 8, 235. 23552117 10.1038/nnano.2013.46

[48] D. K. Sam , H. Li , Y.‐T. Xu , Y. Cao , Carbon 2025, 238, 120214.

[49] S. Nomura , T. Arima , H. Zhao , Z. He , X. Guo , P. Lian , Z. Liu , Phys. Chem. Chem. Phys. 2007, 9, 1276.17347700

[50] D. B. Schuepfer , F. Badaczewski , J. M. Guerra‐Castro , D. M. Hofmann , C. Heiliger , B. Smarsly , P. J. Klar , Phys. Rev. B 2010, 82, 125429.

[51] L. G. Cançado , A. Reina , J. Kong , M. S. Dresselhaus , Phys. Rev. B 2008, 77, 245408.

[52] D. M. Basko , S. Piscanec , A. C. Ferrari , Phys. Rev. B 2009, 80, 165413.

[53] D. Graf , F. Molitor , K. Ensslin , C. Stampfer , A. Jungen , C. Hierold , L. Wirtz , Nano Lett. 2007, 7, 238. 17297984 10.1021/nl061702a

