# Supplementary material for: Lightweight and High‐Strength Carbon Bulk Preparation Via Low‐Temperature Pressureless Sintering of Sugar‐Derived Carbon
Source: Adv Sci (Weinh). 2025 Nov 21;13(7):e18437. doi: 10.1002/advs.202518437 (PMC12866765; doi:10.1002/advs.202518437)
Supplement: Supplementary file 1 — Supporting Information [file ADVS-13-e18437-s001.docx]

Supporting Information

Lightweight and High-Strength Carbon Bulk preparation via Low-Temperature Pressureless Sintering of Sugar-Derived Carbon

Chenlin Hou^1^, Daming Chen^1^, Mingyi Tan^1,*^, Wenzheng Zhang^2,3,*^, Boqian Sun^3^, Guiqing Chen^1^, Wenbo Han^1^, Ruiqun Pan^4^, Shanyi Du^1^, Jiecai Han^1^, Xinghong Zhang^1,2,*^

^1^National Key Laboratory of Science and Technology on Advanced Composites in Special Environments, Harbin Institute of Technology, Harbin 150001, P. R. China;

^2^Suzhou Laboratory, Suzhou 215000, P.R. China;

^3^Jiangsu Mogui New Materials Co., Ltd., Changzhou 213127, P. R. China;

^4^School of Material Science and Chemical Engineering, Harbin University of Science and Technology, Harbin 150001, P. R. China

*Corresponding author. E-mail: zhangxh@hit.edu.cn (Xinghong Zhang); tanmy@hit.edu.cn (Mingyi Tan); zhangwz@szlab.ac.cn (Wenzheng Zhang)

**This file includes:**

Supplementary Figure S1 to S11

Supplementary Table S1 and S2

Reference

**
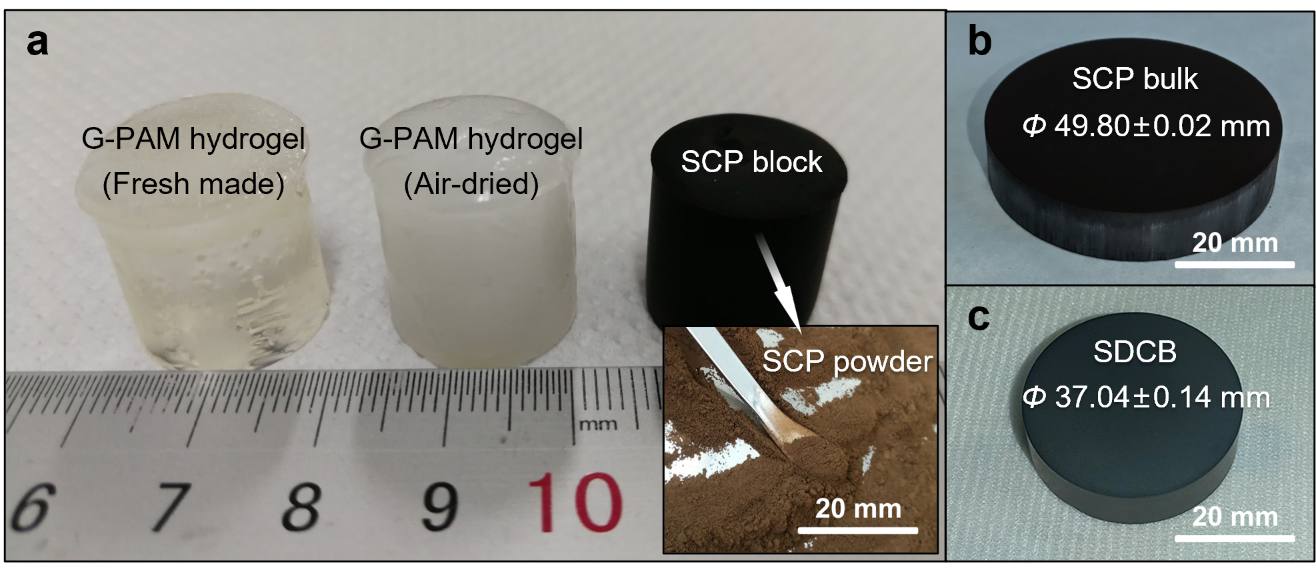
**

**Figure S1.** Photographs of samples at different stages. a) Hydrogels and precursors. b) Compacted SCP bulk. c) SDCB.

**
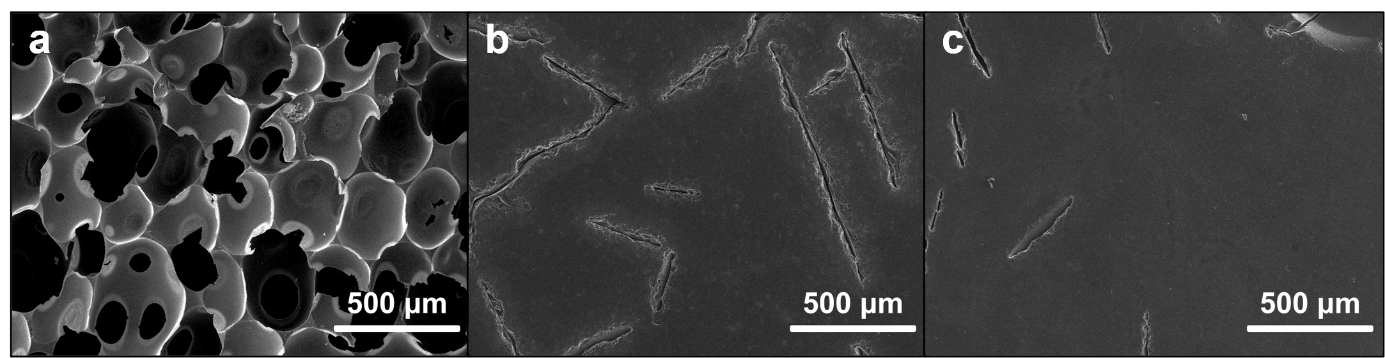
**

**Figure S2.** Cross section of carbonized samples. a) Glucose (foamed). b) G-PAM hydrogel (not foamed). c) SCP block (not foamed).


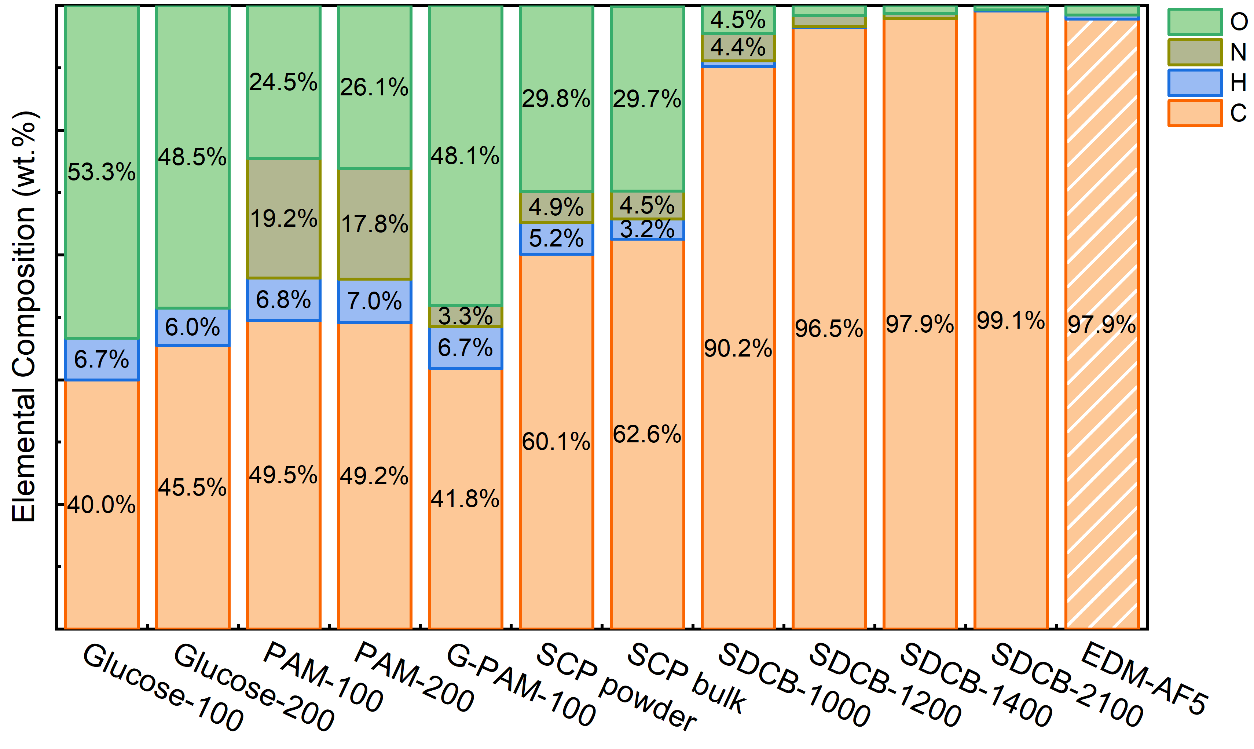


**Figure S3.** Elemental composition measured through organic elemental analysis (OEA).

**
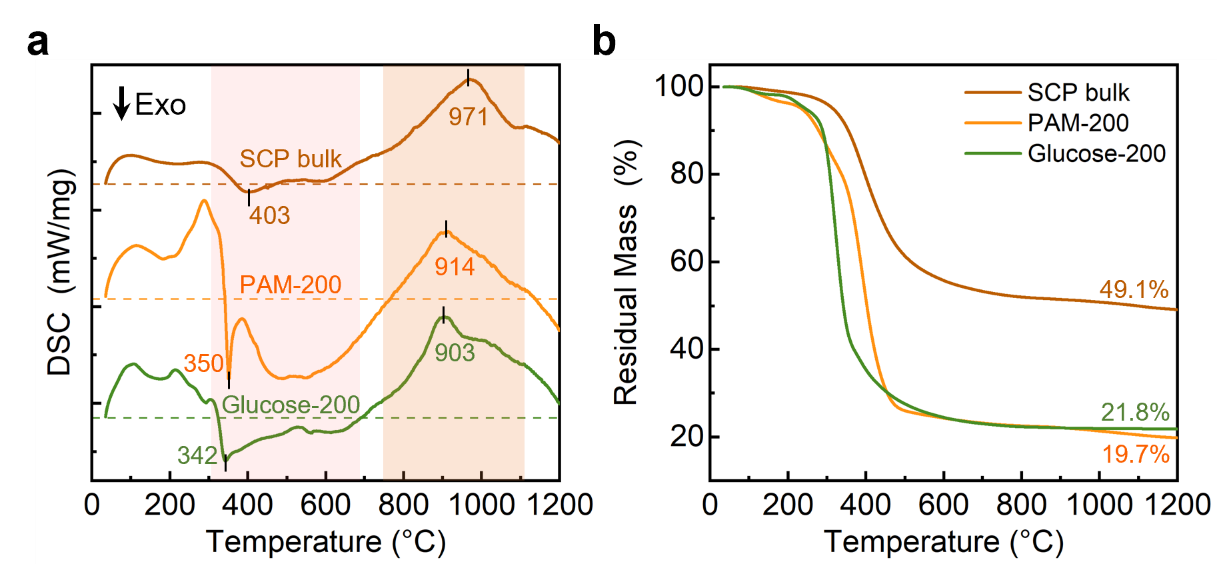
**

**Figure S4.** Thermal analyses: a) DSC curves. b) TG curves.

**
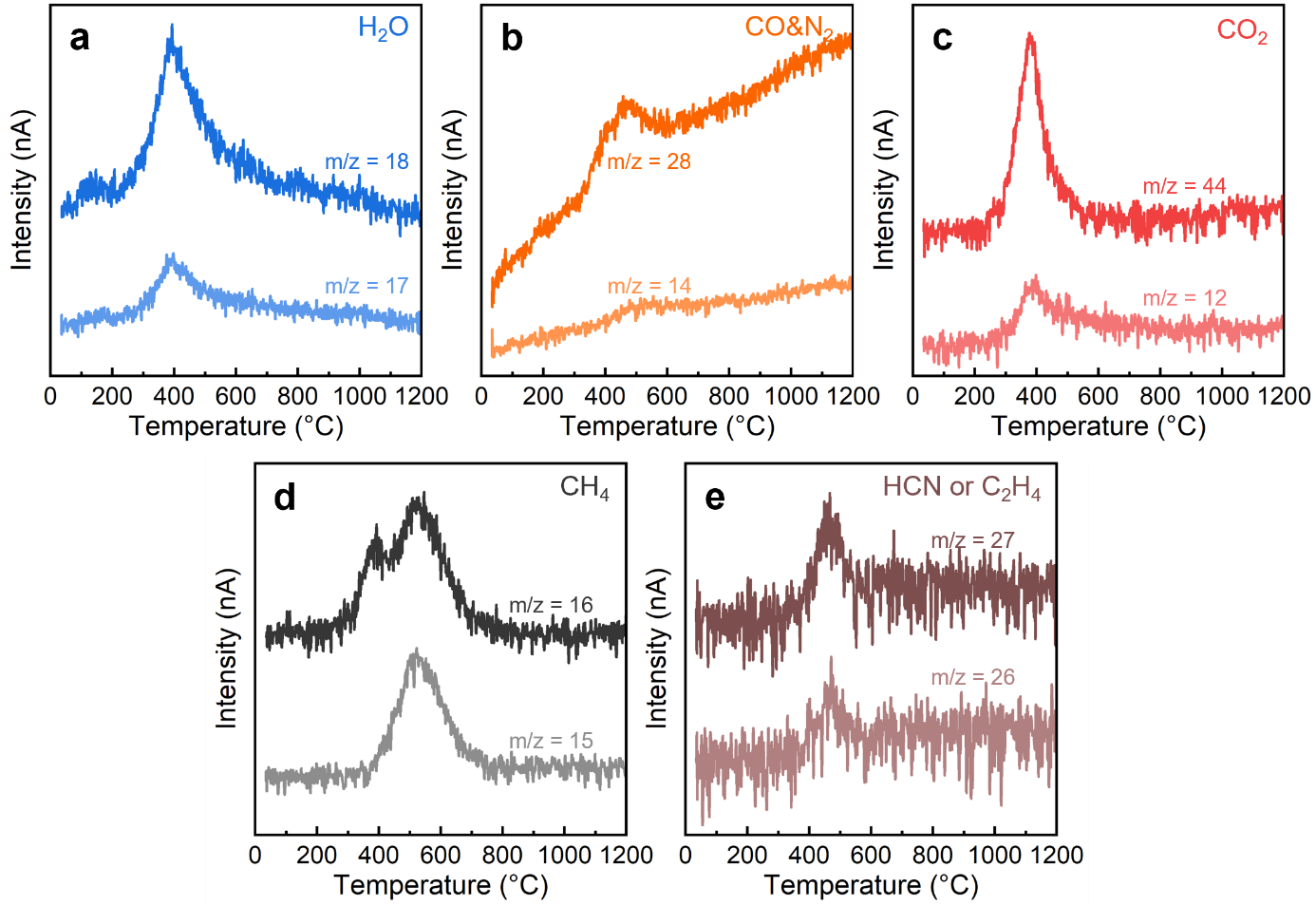
**

**Figure S5.** MS curves of the exhaust from the TG measurement of SCP bulks.
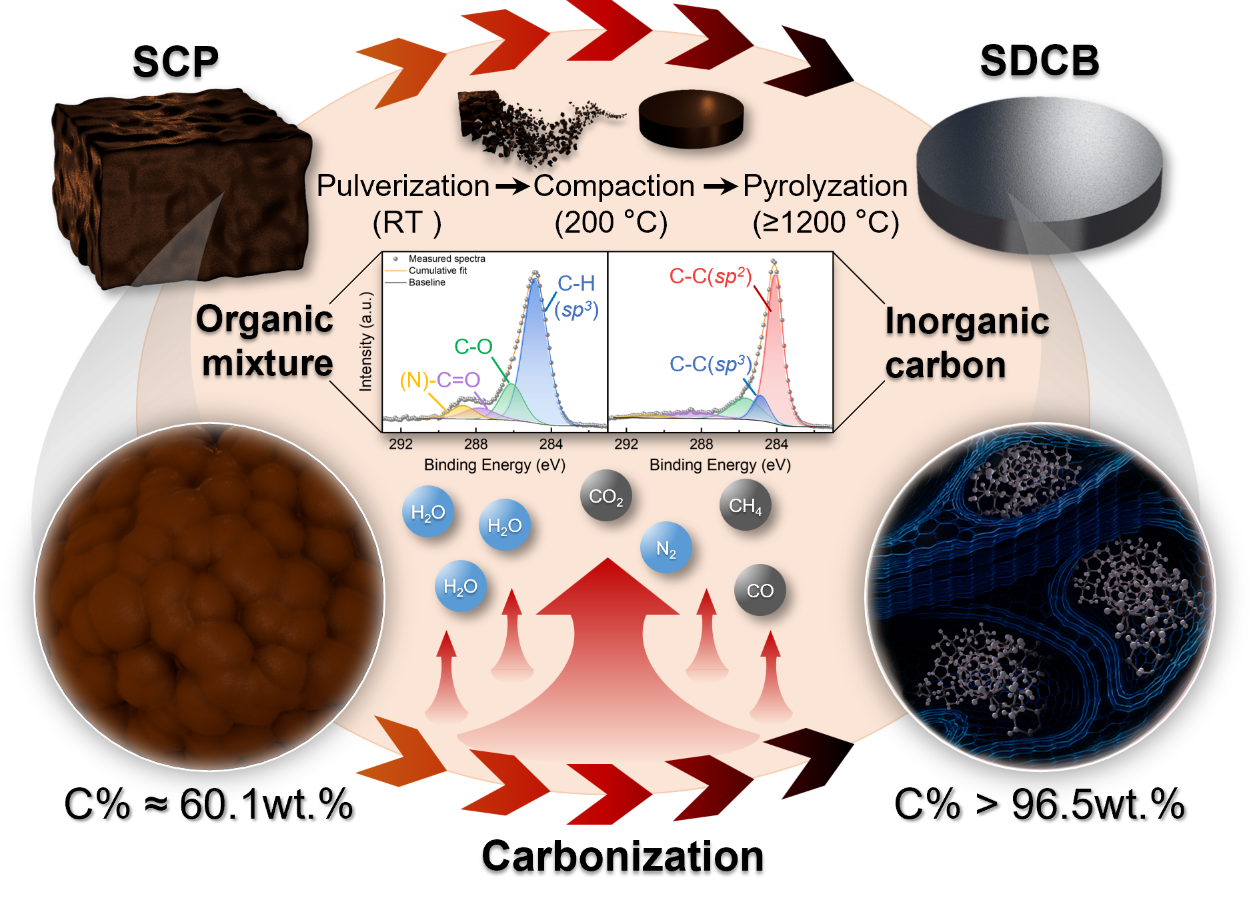


**Figure S6.** The transformation mechanism from SCP to SDCB.


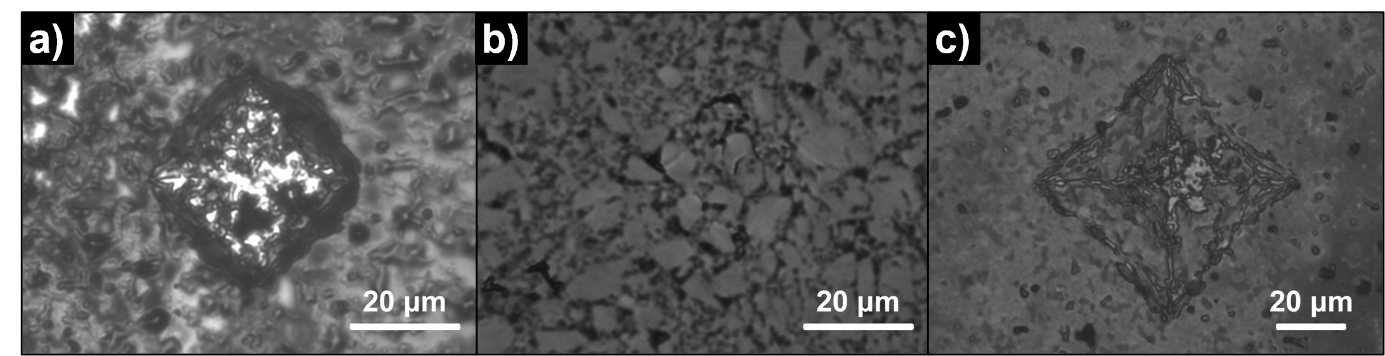


**Figure S7.** Indentation images from micro-Vickers hardness tests: a) SDCB-1200, b) SDCB-2100 (less visible due to rebound), c) EDM-AF5.


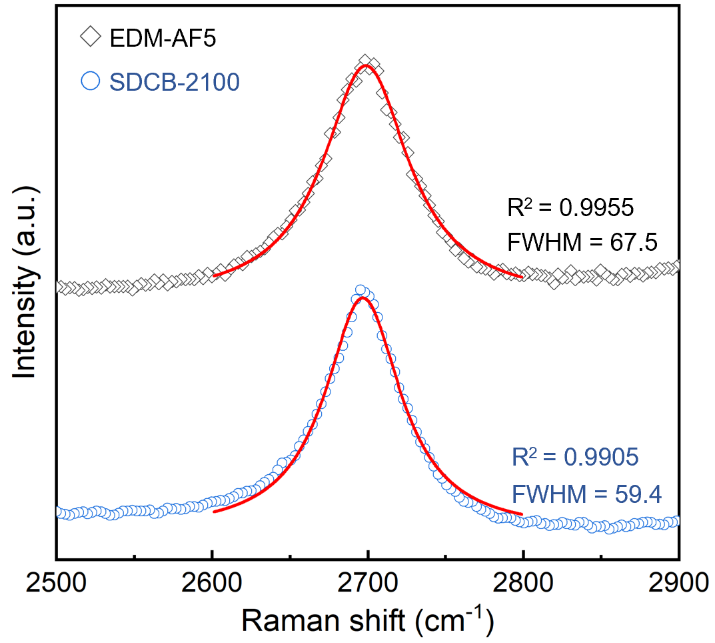


**Figure S8.** Single Lorentzian profiles of the 2D peaks from the Raman curves of EDM-AF5 and SDCB-2100.


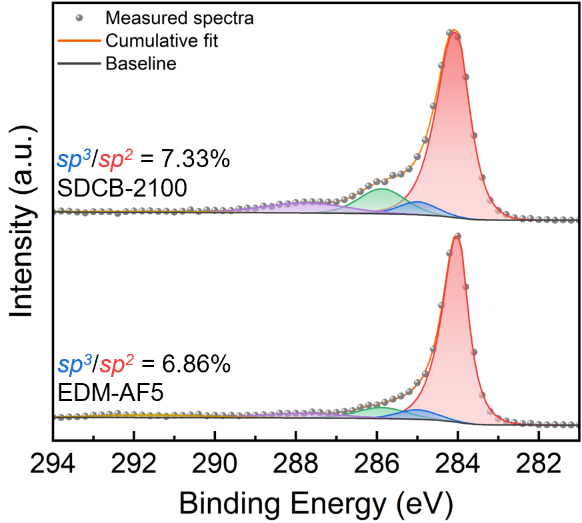


**Figure S9.** XPS C1s of the SDCB-2100 and the EDM-AF5.

**
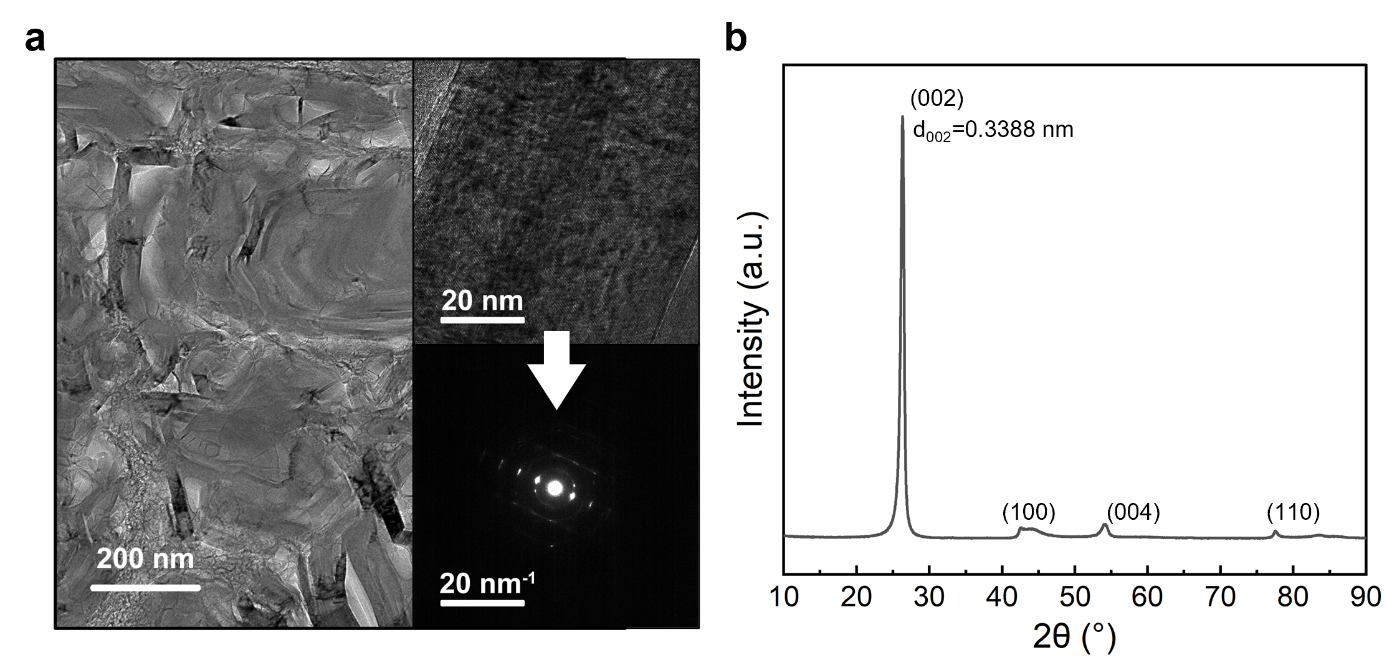
Figure S10.** Microstructures of SDCBs treated at 3000 °C in ambient pressure: a) HRTEM image. b) XRD.


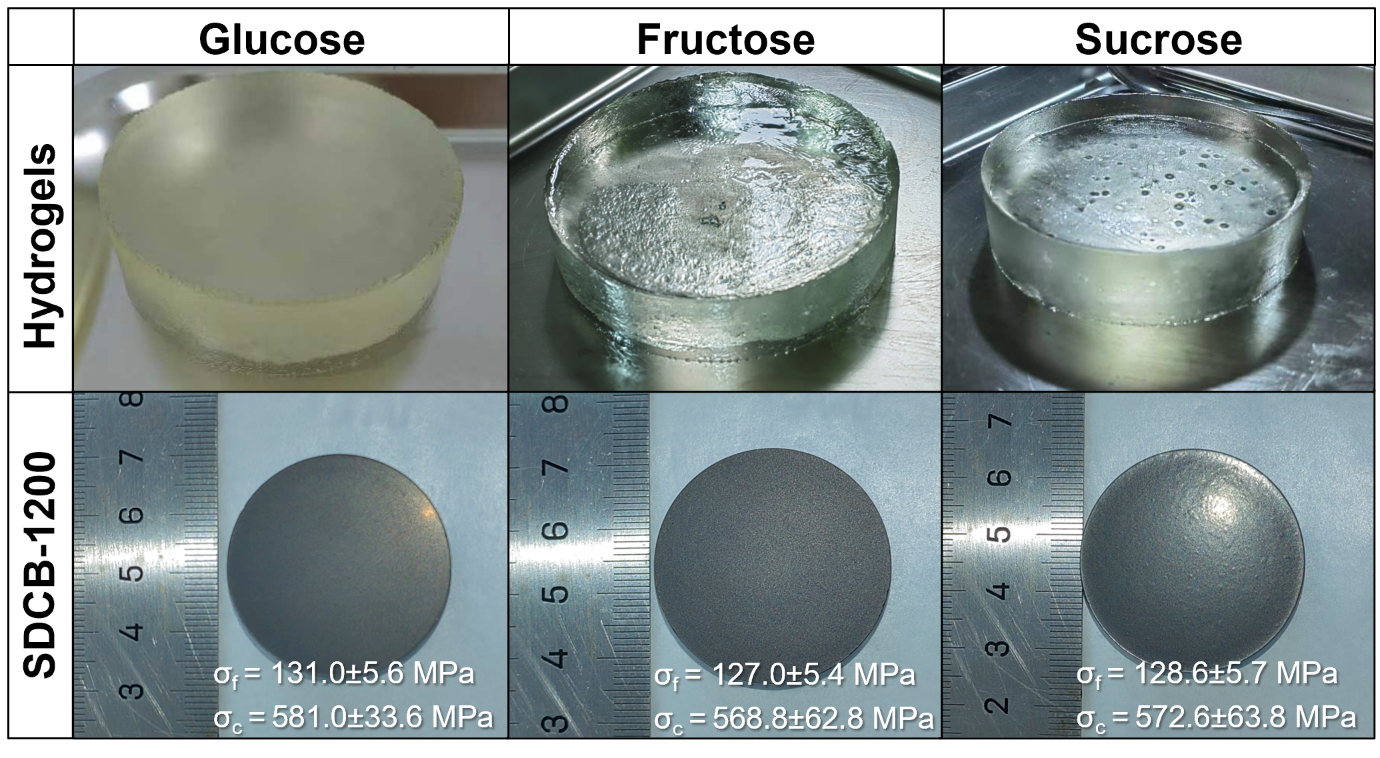


**Figure S11.** Hydrogels and SDCBs fabricated through different sugars.

**Table S1.** Compressive and flexural properties of various grades of bulk carbon materials.

| Bulk carbon materials | Bulk density (g/cm^3^) | Compressive strength (MPa) | Flexural strength (MPa) | Reference |
| --- | --- | --- | --- | --- |
| IG-11 | 1.77 | 78 | 39 | [1] |
| IG-12 | 1.78 | 88 | 39 | [1] |
| IG-15 | 1.90 | 103 | 54 | [1] |
| IG-19 | 1.75 | 88 | 38 | [1] |
| IG-43 | 1.82 | 90 | 54 | [1] |
| IG-45 | 1.88 | 110 | 60 | [1] |
| IG-56 | 1.77 | 88 | 43 | [1] |
| IG-70 | 1.83 | 103 | 47 | [1] |
| ISEM-1 | 1.68 | 69 | 36 | [1] |
| ISEM-2 | 1.78 | 83 | 41 | [1] |
| ISEM-3 | 1.85 | 103 | 49 | [1] |
| ISEM-8 | 1.78 | 106 | 52 | [1] |
| ISO-63 | 1.78 | 135 | 65 | [1] |
| ISO-66 | 1.82 | 134 | 70 | [1] |
| ISO-68 | 1.82 | 172 | 76 | [1] |
| TTK-50 | 1.80 | 130 | 60 | [1] |
| TTK-4 | 1.78 | 135 | 73 | [1] |
| TTK-5 | 1.78 | 150 | 80 | [1] |
| TTK-8 | 1.77 | 155 | 80 | [1] |
| TTK-9 | 1.77 | 180 | 92 | [1] |
| SIC-6 | 1.85 | 103 | 49 | [1] |
| SIC-12 | 1.77 | 93 | 47 | [1] |
| HPG-51 | 1.78 | 140 | 75 | [1] |
| HPG-53 | 1.78 | 156 | 80 | [1] |
| HPG-59 | 1.91 | 210 | 100 | [1] |
| HPG-81 | 1.77 | 161 | 83 | [1] |
| HPG-83 | 1.77 | 187 | 96 | [1] |
| EK20 | 1.70 | 155 | 55 | [2] |
| EK23 | 1.75 | 100 | 40 | [2] |
| EK24 | 1.70 | 180 | 60 | [2] |
| EK40 | 1.70 | 100 | 35 | [2] |
| EK60 | 1.73 | 120 | 80 | [2] |
| EK2209 | 1.77 | 190 | 65 | [2] |
| V1626 | 1.85 | 150 | 58 | [2] |
| Ellor+18 | 1.78 | - | 45 | [3] |
| Ellor+20 | 1.81 | - | 52 | [3] |
| Ellor+25 | 1.83 | - | 55 | [3] |
| Ellor+30 | 1.84 | - | 65 | [3] |
| Ellor+40 | 1.78 | - | 60 | [3] |
| Ellor+50 | 1.86 | - | 76 | [3] |
| Ellor+DS4 | 1.88 | - | 90 | [3] |
| JP4 00 | 1.81 | 100 | 50 | [4] |
| JP5 00 | 1.68 | 155 | 38 | [4] |
| JP5 45 | 1.84 | 140 | 65 | [4] |
| JP5 68 | 2.40 | 165 | 80 | [4] |
| JP6 00 | 1.68 | 125 | 60 | [4] |
| JP6 45 | 1.80 | 165 | 78 | [4] |
| JP6 68 | 2.30 | 168 | 80 | [4] |
| JP9 00 | 1.83 | 120 | 57 | [4] |
| JP9 28 | 1.92 | 130 | 60 | [4] |
| JP9 32 | 1.88 | 120 | 57 | [4] |
| JP9 33 | 1.88 | 120 | 57 | [4] |
| JP9 45 | 1.95 | 160 | 70 | [4] |
| JP9 68 | 2.40 | 155 | 75 | [4] |
| JP10 00 | 1.90 | 210 | 75 | [4] |
| JP10 33 | 1.94 | 170 | 85 | [4] |
| JP13 00 | 1.74 | 115 | 55 | [4] |
| JP13 28 | 1.84 | 115 | 55 | [4] |
| JP13 45 | 1.87 | 160 | 75 | [4] |
| JP13 51 | 1.80 | 115 | 55 | [4] |
| JP13 68 | 2.45 | 185 | 87 | [4] |
| JP19 00 | 1.84 | 230 | 100 | [4] |
| JP19 32 | 1.91 | 210 | 100 | [4] |
| EDM-1 | 1.83 | 98 | 67 | [5] |
| EDM-3 | 1.81 | 125 | 92 | [5] |
| EDM-4 | 1.82 | 148 | 121 | [5] |
| EDM-AF5 | 1.77 | 152 | 100 | [5] |
| EDM-200 | 1.82 | 107 | 62 | [5] |
| EDM-2 | 1.74 | 117 | 77 | [5] |
| ZXF-5Q | 1.78 | 175 | 112 | [6] |
| ACF-10Q | 1.77 | 186 | 97 | [6] |
| AXF-5Q | 1.78 | 138 | 86 | [6] |
| AXM-5Q | 1.73 | 124 | 69 | [6] |
| AXZ-5Q | 1.66 | 103 | 52 | [6] |
| SCB-E | 1.66 | 282.6 | 69.4 | [7] |
| SCB-C | 1.42 | 68.6 | 22.7 | [7] |
| SCB-O | 1.33 | 44.4 | 12.0 | [7] |
| SG-E | 1.75 | 81.2 | 55.9 | [7] |
| SG-C | 1.50 | 31.6 | 13.8 | [7] |
| SG-O | 1.39 | 17.7 | 7.2 | [7] |
| MG-E | 1.94 | - | 100.1 | [7] |
| UHSBC | 1.84 | - | 150 | [8] |
| HSCB-30 | 1.80 | - | 80 | [9] |
| SGA | 1.76 | 91.30 | 45.7 | [10] |
| SGB | 1.84 | 143.50 | 52.4 | [10] |
| SGC | 1.92 | 185.40 | 92.6 | [10] |
| GNC-1 | 2.00 | - | 97 | [11] |
| GNC-5 | 1.99 | - | 108 | [11] |
| GMC-5 | 2.01 | - | 107 | [11] |
| MNC-5 | 1.99 | - | 72 | [11] |
| GNC-10 | 2.02 | - | 73 | [11] |
| GNI-1 | 2.00 | - | 58 | [11] |
| GNI-5 | 2.05 | - | 70 | [11] |
| GNI-10 | 2.08 | - | 33 | [11] |
| YG1 | 1.82 | 124.10 | 78.4 | [12] |
| YG2 | 1.92 | 160.10 | 80.2 | [12] |
| YG3 | 2.01 | 205.20 | 85.8 | [12] |
| YG4 | 2.03 | 208.40 | 102.9 | [12] |
| YG5 | 2.06 | 170.20 | 86.8 | [12] |
| CB-CTP220 | 1.66 | 157.29 | 68.14 | [13] |
| HPHT sintered carbon-graphite blocks | 2.15 | 192 | 87 | [14] |
| PA12-BC10 | - | - | 100.9 | [15] |
| HSG-15 | 1.61 | 445 | 73.6 | [16] |
| CB-2200 | 1.40 | 169 | 69 | [17] |
| TNC-18 | 1.99 | - | 134 | [18] |
| ATG-30 | 1.82 | 131.27 | 54.11 | [19] |
| GBS-WJ | 1.77 | 60.76 | 32.48 | [20] |
| Graphitized artifact | 1.35 | - | 10.3 | [21] |
| C/CoB-1800 | 2.68 | 151.56 | 101.17 | [22] |
| GPC/QI-17-C | 1.56 | 136.98 | 39.61 | [23] |
| WDC-CNTs-PyC | 0.67 | 85.4 | - | [24] |
| Impregnated carbon block | 1.83 | - | 81 | [25] |
| DCMI-C | 1.48 | 54.67 | 17.78 | [26] |
| Graphitized bulk | 1.85 | 46.2 | 22.8 | [27] |
| Graphite blocks 2400 | 1.79 | - | 34.1 | [28] |
| CNFs-0.7 | 1.49 | 60.46 | 36.50 | [29] |

**Table S2.** Properties of the SDCBs and EDM-AF5

|  | Density (g/cm^3^) | | | Compressive strength (MPa) | Flexural strength (MPa) | Micro Vickers hardness (GPa) | Electrical resistance (mΩ·cm) |
| --- | --- | --- | --- | --- | --- | --- | --- |
|  | bulk | skeletal | |  |  |  |  |
| SDCB-1200 | 1.285 | | 1.835 | 581.00 ± 33.61 | 131.00 ± 5.56 | 1.122 ± 0.081 | 4.617 |
| SDCB-2100 | 1.277 | | 1.560 | 361.77 ± 50.05 | 153.41 ± 15.76 | 1.466 ± 0.167 | 5.050 |
| EDM-AF5 | 1.769 | | 2.113 | 181.25 ± 7.074 | 103.34 ± 5.16 | 0.443 ± 0.007 | 1.563 |

References

[1] TOYO TANSO. http://www.sttanso.com/pro_info.aspx?CateId=143.

[2] Sgl carbon, SIGRAFINE® gesenkgepresste Kohlenstoffe und Graphite. https://www.sglcarbon.com/loesungen/material/sigrafine-gesenkgepresste-kohlenstoffe-und-graphite/.

[3] Mersen. https://www.mersengroup.cn/zh-hans/products/isostatic-graphite-extruded-graphite/electrical-discharge-machining-edm.

[4] Mersen. https://www.mersengroup.cn/zh-hans/products/isostatic-graphite-extruded-graphite/mechanical-components-aeronautics.

[5] POCO Materials, Entegris. https://poco.entegris.com/en/home/products/premium-graphite/edm-grades.html.

[6] POCO Materials, Entegris. https://poco.entegris.com/en/home/products/premium-graphite/industrial-grades.html

[7] Wu X, Li R, Tan J et al. Significantly Enhanced Density and Mechanical Strength of Carbon/Graphite Blocks by Waste Gas Pressurized Sintering. *Adv. Mater.* **2025**, *37*, 2505639.

[8] J. Ran, K. Lin, H. Yang, J. Li, L. Wang, W. Jiang, *Appl. Phys. A* **2018**, *124*, DOI 10.1007/s00339-018-1691-5.

[9] B. Miao, J. Wang, J. Li, S. Gu, L. Wang, W. Jiang, *J. Adv. Ceram.* **2022**, *11*, 1815.

[10] H. Zhao, Z. He, X. Guo, P. Lian, Z. Liu, *New Carbon Mater.* **2020**, *35*, 184.

[11] D. K. Sam, H. Li, Y.-T. Xu, Y. Cao, *Carbon* **2024**, *217*, 118630.

[12] L. Zhang, F. Deng, X. Chen, Z. Guo, H. Liu, X. Xing, Z. Zhang, *Diam. Relat. Mater.* **2022**, *128*, 109271.

[13] Y. Chen, S. Gao, P. Gong, P. Liu, J. Tan, X. He, H. Luo, R. Li, C. Tu, *Fuel* **2025**, *401*, 135845.

[14] H. Chen, F. Deng, X. Xing, X. Chen, Z. Liu, W. Deng, J. Yu, C. Li, Z. Ye, Y. Lai, B. Lv, *Ceram. Int.* **2025**, *51*, 39620.

[15] H. Baniasadi, T. Puttonen, R. Abidnejad, S. Jayaprakash, J. Partanen, E. Lizundia, J. Niskanen, *Chem. Eng. J.* **2025**, *519*, 165502.

[16] Q. Zhang, J. Fang, W. Ma, A. Li, Q. Liu, R. Chang, Q. Li, S. Jia, B. Liu, Y. Zhang, Y. Mu, Q. Huang, *Diam. Relat. Mater.* **2025**, *157*, 112518.

[17] M. Hu, J. Pang, Y. Tang, M. Ma, J. Huang, P. Li, L. Shi, Y. Zhong, J. He, J. Ding, *Inorg. Chem.* **2025**, *64*, 11786.

[18] S. Gu, H. Chen, J. Wang, X. Lu, L. Wang, W. Jiang, *New Carbon Mater.* **2025**, *40*, 703.

[19] P. Gong, C. Li, J. Tan, P. Liu, X. He, Y. Chen, X. Zeng, R. Li, C. Tu, *Fuel* **2025**, *387*, 134338.

[20] S. Li, H. Wu, C. Huang, S. Zeng, L. Gong, X. Ye, H. Zhang, A. Gao, M. Liu, *Mater. Sci. Eng. B* **2025**, *314*, 118071.

[21] X. Cao, S. Lv, H. Lan, H. Liu, H. Chen, J. Liu, T. Ouyang, K. Shen, *Carbon* **2025**, *234*, 120029.

[22] Y. Zhang, J. Wang, Y. Zhang, Q. Zheng, L. Wang, W. Jiang, *Adv. Sci.* **2025**, *12*, 2416210.

[23] J. Tan, P. Gong, X. Wu, P. Liu, G. Ren, Y. Qu, R. Li, C. Tu, *Fuel Process. Technol.* **2024**, *260*, 108103.

[24] B. Zhang, L. Zhang, Z. Wang, Q. Gao, *J. Mater. Sci. Technol.* **2024**, *175*, 22.

[25] W. Kanlayakan, S. Lhosupasirirat, C. Amnatsin, N. Verojpipath, B. Pragobjinda, T. Srikhirin, *ACS Omega* **2023**, *8*, 27312.

[26] J. Tan, P. Liu, P. Gong, Y. Liu, J. Chen, R. Li, C. Tu, *Fuel* **2023**, *343*, 127912.

[27] L. Zhong, L. Guo, J. Wang, Q. Song, H. Li, Y. Li, *Carbon* **2023**, *208*, 123.

[28] S.-H. Lee, Y.-M. Hwang, T.-S. Byun, J.-H. Ko, J.-S. Roh, *Carbon* **2023**, *208*, 443.

[29] Y. Chen, C. Tu, Y. Liu, P. Liu, P. Gong, G. Wu, X. Huang, J. Chen, T. Liu, J. Jiang, *Carbon Lett.* **2023**, *33*, 561.
